# The Diguanylate Cyclase HsbD Intersects with the HptB Regulatory Cascade to Control *Pseudomonas aeruginosa* Biofilm and Motility

**DOI:** 10.1371/journal.pgen.1006354

**Published:** 2016-10-28

**Authors:** Martina Valentini, Benoît-Joseph Laventie, Joana Moscoso, Urs Jenal, Alain Filloux

**Affiliations:** 1 MRC Centre for Molecular Microbiology and Infection, Department of Life Sciences, Imperial College London, London, United Kingdom; 2 Focal area of Infection Biology, Biozentrum, University of Basel, Basel, Switzerland; Max Planck Institute for Terrestrial Microbiology, GERMANY

## Abstract

The molecular basis of second messenger signaling relies on an array of proteins that synthesize, degrade or bind the molecule to produce coherent functional outputs. Cyclic di-GMP (c-di-GMP) has emerged as a eubacterial nucleotide second messenger regulating a plethora of key behaviors, like the transition from planktonic cells to biofilm communities. The striking multiplicity of c-di-GMP control modules and regulated cellular functions raised the question of signaling specificity. Are c-di-GMP signaling routes exclusively dependent on a central hub or can they be locally administrated? In this study, we show an example of how c-di-GMP signaling gains output specificity in *Pseudomonas aeruginosa*. We observed the occurrence in *P*. *aeruginosa* of a c-di-GMP synthase gene, *hsbD*, in the proximity of the *hptB* and flagellar genes cluster. We show that the HptB pathway controls biofilm formation and motility by involving both HsbD and the anti-anti-sigma factor HsbA. The rewiring of c-di-GMP signaling into the HptB cascade relies on the original interaction between HsbD and HsbA and on the control of HsbD dynamic localization at the cell poles.

## Author Summary

One of the most complex c-di-GMP signaling systems is found in the human opportunistic pathogen *Pseudomonas aeruginosa*. The intracellular concentration of c-di-GMP determines both bacterial physiology and pathogenesis. Here, we highlight the complexity of c-di-GMP-dependent signaling routes by giving the example of the HsbD diguanylate cyclase. This protein, which synthesizes c-di-GMP, evolved in *P*. *aeruginosa* to locally implement the HptB regulatory pathway for the control of swarming, twitching, swimming motilities and biofilm formation. Understanding how the pleiotropic molecule c-di-GMP targets a particular bacterial process is equivalent to understand how bacteria translate signal information into specific actions. This knowledge is ultimately relevant for novel medical treatments aiming to block these actions in order to prevent the successful establishment of a bacterial infection.

## Introduction

In both eukaryotes and prokaryotes cyclic nucleotides are key intracellular signaling molecules that are able to rapidly amplify environmental signals and translate them into distinct cellular outputs. How these signaling systems reach outcome specificity when controlling numerous functions is still an unresolved issue. It is proposed to be achieved mainly through a temporal and spatial control of the second messenger levels [1–3]. In the bacterial world, cyclic di-GMP (c-di-GMP) signaling has gained momentum since it has been proposed to be universal and pleiotropic (*i*.*e*. it regulates a wide range of functions from virulence, to motility, cell cycle and biofilm formation) [2, 4, 5]. Recently c-di-GMP signaling also entered the eukaryotic field as it locally induces stalk cell differentiation in *Dictyostelium discoideum* [6]. Additionally, c-di-GMP is recognized as a microbial molecule by the innate immune system of mammalian cells via the STING sensor, which consequently activate a STING-dependent type I IFN response [7].

Despite the complexity and diversity of c-di-GMP regulated processes, a fundamental hallmark is its role in determining the bacterial lifestyle: high levels fostering sessility while low levels favoring the planktonic state [5, 8, 9]. The c-di-GMP levels are modulated in the cell by two classes of proteins: diguanylate cyclases (DGCs) and phosphodiesterases (PDEs). The former carry a GGDEF-containing domain that is involved in the synthesis of c-di-GMP from GTP molecules; the latter contain a domain with EAL or HD-GYP motifs that hydrolyses c-di-GMP producing pGpG or GMP [10]. Most of these enzymes carry receiver or transmission domains, such as those found in two component regulatory systems, suggesting that their action can be modulated by environmental signals or by their integration into specific bacterial regulatory circuits [8, 11, 12].

The *Pseudomonas aeruginosa* genome comprises a high number of genes (43) coding for GGDEF, EAL, HD-GYP proteins [13–15]. Interestingly, these enzymes do not appear to be redundant and have specific impact on biofilm formation or cytotoxicity [16]. The c-di-GMP is a small molecule and presumably freely diffusible in the bacterial cytoplasm. How can a single DGC faithfully transmit this information (c-di-GMP) and trigger only a subset of c-di-GMP regulated behaviors, thus avoiding undesired cross-talk? Here, we give an original example of how a DGC achieve specificity of action through the interaction with a molecular network regulating important *P*. *aeruginosa* behaviors, namely the HptB pathway. The HptB pathway controls biofilm formation, twitching, swimming, swarming motility and chemotaxis in *P*. *aeruginosa* by partially interfering with flagellar gene expression and by partially intersecting with the Gac/Rsm cascade [17–20]. It involves the HsbR response regulator and the HsbA anti-anti sigma factor, as outlined in S1 Fig. Central in the HptB pathway is the HsbA phosphorylation state, which determines a switch in partner for HsbA. Briefly, when HptB is phosphorylated, it activates the HsbR phosphatase domain, which in turn de-phosphorylates HsbA. HsbA subsequently binds the anti-sigma factor FlgM, inducing flagellar gene expression. In absence of HptB, or when the protein is not phosphorylated, HsbR acts as a kinase and phosphorylates HsbA (HsbA-P). This switch results in a decrease of swarming motility while an increase of biofilm formation is observed [17, 18]. The control of *P*. *aeruginosa* biofilm formation by the HptB pathway occurs *via* a reduction in the levels of the RsmY small regulatory RNA (sRNA) [17]. In *P*. *aeruginosa* the RsmY and RsmZ sRNAs are core component of the Gac/Rsm cascade together with the GacS/GacA two-component system and the RsmA translational repressor [21]. Upon activation the GacS/GacA two-component system induces expression of the RsmY and RsmZ sRNA-encoding genes, which sequester the mRNA-binding protein RsmA. Titration of RsmA leads to production of biofilm determinants, whilst free RsmA correlates with a planktonic/virulent lifestyle [21, 22]. Additional regulators modulate the Gac/Rsm system, notably the RetS and LadS hybrid sensors [23].

Our laboratory has previously uncovered a connection between c-di-GMP signaling and the Gac/Rsm pathway for controlling the switch between “planktonic/virulent” and “sessile/biofilm” behaviors in *P*. *aeruginosa* [24]. The link has been later on elucidated in molecular details: SadC, a DGC which production is repressed by RsmA, is a central player for the Gac/Rsm regulation of biofilm formation. Precisely, the hyper-biofilm phenotype and high c-di-GMP levels observed when mutating some components of the Gac/Rsm cascade, could be brought back to wild-type levels by the additional deletion of *sadC* [25].

Here, we characterize a new DGC, HsbD, which regulates twitching, swarming, chemotaxis and biofilm formation by relaying information within the HptB pathway and therefore also into the Gac/Rsm pathway. We provide clues about how *P*. *aeruginosa* evolved and developed this new network by acquiring the *hsbD* gene and by relocating the *hptB* cluster in a genomic region next to some flagellar genes (among which *flgM*). Finally, the regulation of HsbD presented in this paper provides insights into how c-di-GMP signaling can be orchestrated in bacteria to reach output specificity.

## Results

### The *hptB* gene cluster merged with flagellar and chemotaxis genes to evolve into a novel *P*. *aeruginosa* specific flagellar locus

**Fig 1:**
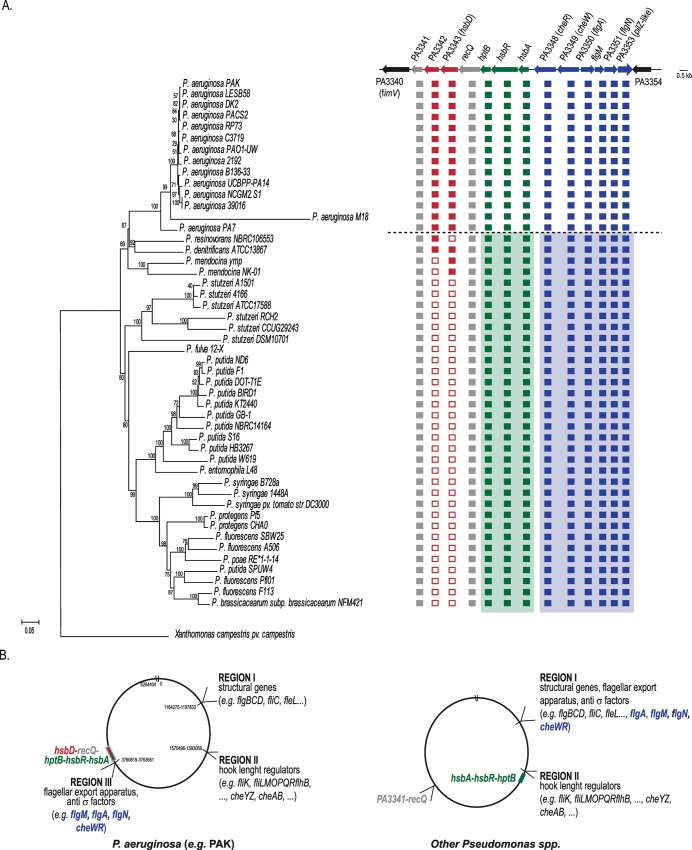
Occurrence of PA3343 (*hsbD*) in *Pseudomonas* strains and its relation with the flagellar genes reorganization in *P*. *aeruginosa*. **(A)** Evolutionary relationships of *Pseudomonas* taxa carrying *hptB* orthologs and taxonomic distribution of PA3341-PA3353 genes. The phylogenetic tree of *Pseudomonas* strains was constructed using MEGA 6 [58]. The percentage of replicate trees in which the associated taxa clustered together in the bootstrap test (1000 replicates) is shown next to the branches. The tree is drawn to scale, with branch lengths in the same units as those of the evolutionary distances used to infer the phylogenetic tree. Filled squares indicate the presence of an ortholog gene, while empty squares indicate the absence. Location of the PA3341-PA3353 genes is shown as in *P*. *aeruginosa*. When a green or blue background is used, it indicates that each gene set is present, but at different location on the chromosome, as explained in panel B. Non-aeruginosa strains are separated by a dashed line. **(B)** Location of flagella and *hptB* related genes in *Pseudomonas*. In *P*. *aeruginosa* (left) flagella genes are located in three regions of the chromosome [68] while in other *Pseudomonas* species (right) two regions are present and in general physically separated with some exceptions (*e*.*g*. *Pseudomonas entomophila* L48) [26].

Our laboratory showed previously the regulatory interactions between HptB (PA3345), the response regulator HsbR (PA3346) and the anti-anti sigma factor HsbA (PA3347) [17]. All three corresponding genes clustered in *Pseudomonas* and are part of an operon [18]. Several upstream sensors have been proposed for the HptB pathway, including PA1611, ErcS’ (PA1976) and SagS (PA2824) [26–28]. The genes encoding these proteins are located at different chromosomal loci. A phylogenetic analysis performed with sequenced *Pseudomonas* strains revealed that all species carry *hsbA/hsbR/hptB* orthologs (Fig 1A). In non-*aeruginosa* species though, these genes are present in the so-called flagella biosynthesis region II, which for example contains genes like *fliK*, encoding the hook length regulators (Fig 1B, right panel). Instead, in *P*. *aeruginosa* the *hsbA/hsbR/hptB* genes relocated to a newly formed flagella biosynthesis region (region III) in the vicinity of the PA3344 (*recQ*) gene, together with flagellar and chemotaxis genes from region I, including the gene encoding the anti-sigma factor FlgM and the two chemotactic genes *cheW/cheR* (Fig 1B, left panel). Given the observed interaction between HsbA and FlgM, this rearrangement is likely functionally relevant [18]. We therefore propose that the *hsbA/hsbR/hptB* genes could be considered as part of the flagella biosynthesis region III.

### Acquisition of a diguanylate cyclase-encoding gene (PA3343) upstream the flagellar region III

In the evolution of *Pseudomonas* strains, and preceding the formation of the flagellar region III, the PA3343 gene seems to have appeared upstream of *recQ* (Fig 1A). The gene is present in all sequenced *P*. *aeruginosa* strains and also in the last common ancestor between *P*. *aeruginosa* and *Pseudomonas mendocina* NK-01, while *Pseudomonas resinovorans* NBRC106553 may have subsequently lost the gene. The PA3342 gene has probably arrived subsequently to PA3343, in the last common ancestor between *P*. *aeruginosa* and *Pseudomonas denitrificans*, just before *P*. *aeruginosa* speciation. Finally, the *recQ* and PA3341 genes appeared to be ancestral genes, being present in the same location in all *Pseudomonas* strains analyzed [29]. The PA3343 gene encodes a putative diguanylate cyclase while PA3342 is of unknown function. Given its location, and our recent work on the importance of c-di-GMP for the Gac/Rsm cascade and the HptB pathway, we decided to investigate the role of PA3343 and to test whether it had an integrated function in the HptB pathway. Based on the results presented below, PA3343 is linked to the HptB cascade; hence, we named the gene *hsbD* (where Hsb stands for “HptB-dependent secretion and biofilm”).

### The HsbD diguanylate cyclase is instrumental to the HptB-dependent regulation of biofilm formation

**Fig 2:**
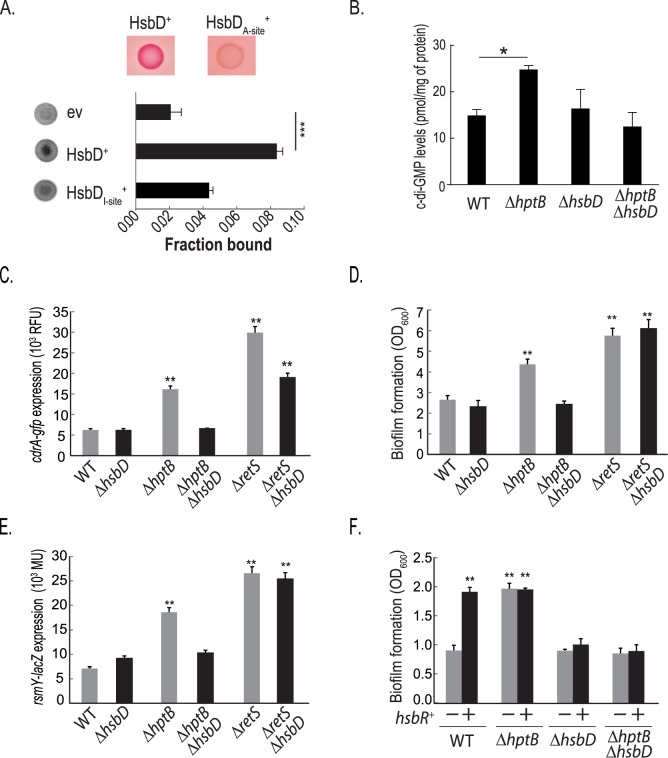
HsbD is a diguanylate cyclase which activity intersects with the HptB regulatory pathway. **(A)** Top panels show *E*.*coli* expressing HsbD and a HsbD variant with a mutated active site (A-site) and detection of DGC activity by Congo red binding. Lower graph shows detection of HsbD binding to ^32^P-labeled c-di-GMP using DRaCALA performed with *E*. *coli* extracts prepared from strains carrying cloning vector (ev), or recombinant plasmids overexpressing His-HsbD, or His-HsbD variant with a mutated inhibitory site (I-site). In all strains the expression and stability of HsbD and variants was prior verified by SDS-page and Western blot. The chart shows the quantification of the fraction of ^32^P-c-di-GMP bound to the protein spot on the nitrocellulose membrane from three independent experiments (Student t-test, ***, *p ≤ 0*.*0001*). **(B)** Graph depicting quantification of c-di-GMP levels in *P*. *aeruginosa* PAK wild type (WT) and PAK Δ*hsbD*, Δ*hptB*, Δ*hptB*Δ*hsbD* mutant strains by LC-MS/MS. Data are expressed as picomoles of c-di-GMP per mg of total protein (see Materials and Methods). Each value is the average of three different cultures ± standard deviation (Student t-test, *, *p* < 0.05). **(C-E)** Experiments performed in *P*. *aeruginosa* PAK wild type (WT) and PAK Δ*hsbD*, Δ*hptB*, Δ*hptB*Δ*hsbD*, Δ*retS* and Δ*retS*Δ*hsbD* mutant strains. **(C)** Expression of a *cdrA*-*gfp* reporter fusion (indicative of c-di-GMP levels) measured in *P*. *aeruginosa* strains grown to OD_600_ ~2.0 in LB medium. Relative fluorescence units (RFU) are corrected for background (empty vector) and for cell density as described in the Materials and Methods. **(D)** Biofilm formation of *P*. *aeruginosa* strains measured by crystal violet staining. Bacterial strains were grown in LB medium in 24-microtiter plates for 14 hours. **(E)** β-Galactosidase activity of a *rsmY–lacZ* transcriptional fusion in *P*. *aeruginosa* strains grown in rich LB medium to an OD600~2.0, as described in Bordi *et al*. (2010). **(F)** Biofilm formation, measured by crystal violet staining, of PAK WT, Δ*hptB*, Δ*hsbD* and Δ*hptB*Δ*hsbD* strains grown in 24-microtiter plates for 8 hours; carrying either a pMMBRMCS4 empty plasmid (grey columns, -) or the pBBR3347 plasmid overexpressing *hsbR* (black columns, +). Each value is the average of three different cultures ± standard deviation (Student t-test, **, *p* < 0.01).

The *hsbD* gene encodes a 389 aa protein that displays a GGDEF domain at the C terminus (252–389 aa) while six transmembrane domains are predicted within the N-terminal region of the protein (52–206 aa) [29]. PA3343/HsbD has previously been described as an enzymatically active diguanylate cyclase *in vitro*, with 445 pmol of c-di-GMP produced per mg of wet cell weight [16]. In order to confirm this observation *in vivo*, an *Escherichia coli* strain overexpressing *hsbD* was grown on solid medium supplemented with the Congo-Red dye (Fig 2A, top panel). As expected, HsbD causes the red colony staining, phenotype correlated with a global increase in intracellular c-di-GMP levels [30, 31]; while a catalytic inactive GGAAF mutant does not trigger such output. The GGDEF domain of HsbD has the conserved RxxD motif (293–296 aa), which forms the allosteric inhibitory site (I-site) of diguanylate cyclases [31]. Binding of c-di-GMP to HsbD was determined by differential radial capillary action of ligand assay (DRaCALA) using ^32^P-labeled c-di-GMP and *E*. *coli* extracts prepared from strains overexpressing either *hsbD* or an *hsbD* variant with a point mutation at the I-site (R_293_➔A, Fig 2A). Significant c-di-GMP binding was observed with the construct encoding wild-type HsbD, when compared to the negative control, while it is drastically reduced with the HsbD I-site mutant.

In order to investigate the impact of HsbD on the HptB pathway, we engineered an *hsbD* deletion in the *P*. *aeruginosa* wild-type and Δ*hptB* (*hptB* deletion) background and tested several phenotypes controlled by the HptB network. First, we followed up on our previous observation that in an *hptB* mutant c-di-GMP levels are high [25] and we monitored variation in c-di-GMP levels by direct measurement of c-di-GMP *via* LC-MS/MS and by using the c-di-GMP responsive *cdrA-gfp* reporter [32]. In the Δ*hptB*Δ*hsbD* background, both c-di-GMP levels and the expression of the *cdrA-gfp* fusion are reduced when compared to the Δ*hptB* background and they are similar to the wild-type strain (Fig 2B and 2C). Concomitantly, we did not observe differences in c-di-GMP levels or in the expression of the reporter construct between a Δ*hsbD* mutant and the wild-type strain. Likely, these results raise the possibility that in the conditions tested HptB negatively regulates HsbD DGC activity, which becomes detectable when *hptB* is deleted. We also observed a reduction of *cdrA* expression by introducing an *hsbD* mutation in the Δ*retS* (*retS* deletion) mutant; although in this genetic context HsbD has a significantly weaker impact than in the Δ*hptB* background. The effect can be explained by the capacity of RetS to transfer the phosphoryl group to HptB [18]. In agreement with this, we observed that introduction of the *hsbD* mutation in the Δ*retS* background does not abrogate the hyper-biofilm phenotype, while it does in the *hptB* mutant (Fig 2D). Finally, complementation of the *hsbD* deletion restored the hyper-biofilm phenotype of the *hptB* mutant (S2 Fig).

The levels of the sRNA RsmY have been previously shown to be increased in an *hptB* mutant [17]. Again, the introduction of the *hsbD* mutation in the Δ*hptB* background abrogates the HptB-dependent (but not the RetS) down-regulation of RsmY levels (Fig 2E), while the RsmZ levels are not affected (S3 Fig). Besides, we showed previously that HsbR acts downstream of HptB and that overexpression of *hsbR* resulted in increase in biofilm formation [17]. In contrast, biofilm formation was not increased when overexpressing *hsbR* in a Δ*hsbD* background (Fig 2F). Thus, *hsbD* is epistatic to *hptB* and *hsbR* with HsbD activity being necessary for the biofilm phenotype of an *hptB* mutant. Altogether, these data revealed that the HsbD DGC is tightly interlinked with the HptB pathway to regulate biofilm formation.

### HsbD interacts with the anti-anti-sigma factor HsbA

**Fig 3:**
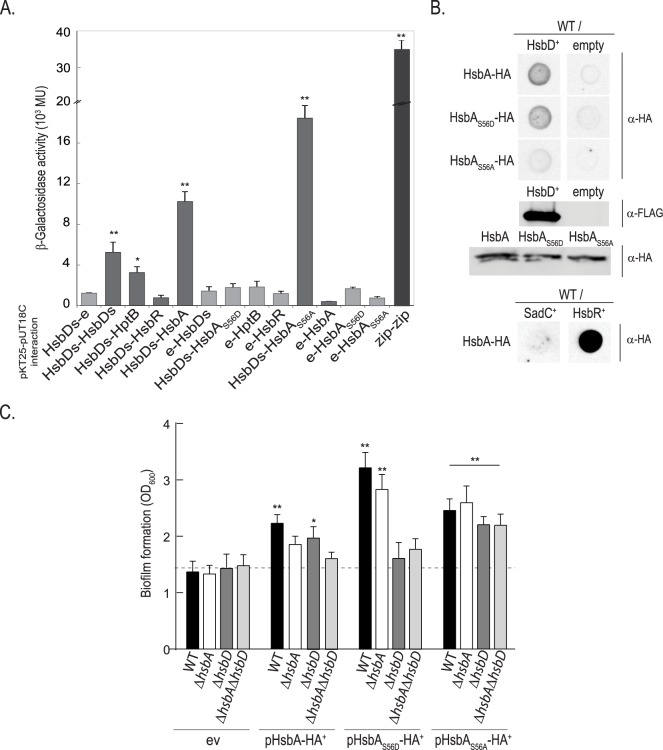
Interaction of HsbD with HsbA is dependent on HsbA phosphorylation. **(A)** Reconstitution of adenylate cyclase in the *E*. *coli* strain DHM1 using a bacterial two-hybrid approach (see S1 Table) was detected by blue staining due to X-gal hydrolysis when colonies where grown on LB–X-gal agar plates containing 0.5 mM IPTG, 100 μg/ml ampicillin, and 50 μg/ml chloramphenicol agar plates. The interactions were also quantified by β-galactosidase assays using liquid cultures of the same strains. Each value is the average of three different cultures ± standard deviation (*, *p* < 0.05; **, *p* < 0.01). **(B)** Dot blot analysis of HsbD-HsbA interaction in *P*. *aeruginosa* PAK. PAK wild type (WT) cell lysates overexpressing HsbD (FLAG-tagged), SadC, HsbR (prey proteins) are spotted on a membrane and incubated with purified HA-tagged HsbA, HsbA_S56D_ and HsbD_S56A_ proteins (bait proteins). Detection of HsbA bait proteins bound to HsbD prey on the blot was performed using α-HA antibody. Empty vector was used as negative control. Production of HsbD-FLAG/HsbA-HA variants is shown by Western blot. **(C)** Biofilm formation of *P*. *aeruginosa* wild-type PAK, Δ*hsbA*, Δ*hsbD* and Δ*hsbA*Δ*hsbD* strains measured by crystal violet staining. Bacterial strains were expressing in *trans* either HsbA, HsbA_S56D_ or HsbA_S56A_ and grown in LB medium in 24-microtiter plates for 14 hours in presence of 50 μg/ml gentamycin and 0.5 mM IPTG. ev: empty vector (pME6032, see S1 Table). Each value is the average of three different cultures ± standard deviation. Asterisks indicate statistically significant difference of biofilm formation compared to the ev (*, *p*< 0.05; **, *p*< 0.01).

The epistatic studies described above indicated that HsbD action intersects with the HptB pathway. We further investigated whether HsbD had direct functional implications in the HptB pathway in terms of protein-protein interaction (Fig 3). We first performed a systematic bacterial two-hybrid (BTH) analysis between HsbD and any putative partners in the HptB signaling cascade (Fig 3A). In order to perform the screen we focused on the cytoplasmic DGC domain of HsbD (HsbDs). While HsbDs interacts with itself (DGCs are dimeric enzymes [33]) no interaction was found with HsbR. Instead, HsbDs interacts with the anti-anti-sigma factor HsbA. Importantly this interaction is lost when the HsbA phosphorylation site, Ser56, is substituted with an alanine. In contrast, a phosphorylated mimicry of HsbA, engineered by replacing Ser56 with an aspartate residue, interacts more strongly with HsbDs, suggesting that phosphorylation of HsbA possibly strengthens the stability of the HsbDs/HsbA complex (Fig 3A). Our screen also identified a weak interaction between HptB and HsbD (Fig 3A). We further tested the HsbD/HsbA interaction in the original *P*. *aeruginosa* host and used a different approach. A blot overlay analysis was performed using cell lysates (Fig 3B) from *P*. *aeruginosa* strains producing or not HsbD, and various forms of the purified HsbA protein tagged with an HA epitope. This experiment confirmed the HsbA-HsbD interaction and its dependency on the HsbA phosphorylation state. While the HsbA-HsbD, HsbAS56D-HsbD and HsbA-HsbR interaction is observed, HsbA does not interact with another DGC, namely SadC, and only a weak signal is detected with the HsbAS56A variant (Fig 3B). All together, these results support the conclusion that HsbD binds to the phosphorylated form of the anti-anti-sigma factor HsbA (HsbA-P). Both *hsbA* and *hsbD* are epistatic to *hptB* for the regulation of biofilm formation (S4 Fig). In order to determine whether HsbD is required for HsbA functionality, we tested the impact of *hsbA* overexpression on biofilm formation in the wild-type PAK, Δ*hsbA*, Δ*hsbD* and Δ*hsbA*Δ*hsbD* strains (Fig 3C). Overexpression of *hsbA* readily increases biofilm formation in the wild-type PAK strain [17]. Given that the interaction of HsbA with HsbD is dependent on the HsbA phosphorylation state, we reasoned that we could separate the phenotypes due to two forms of HsbA (phosphorylated and unphosphorylated). Therefore we tested plasmids carrying *hsbA* versions that mimicked either the phosphorylation or non-phosphorylation states, *i*.*e*. pHsbA_S56D_ or pHsbA_S56A_, respectively. Both constructs were able to increase biofilm formation in the wild-type strain when compared to the strain carrying an empty vector. Interestingly, in the Δ*hsbD* and Δ*hsbA*Δ*hsbD* strains, overproduction of HsbA_S56D_ failed to display the hyper-biofilm phenotype indicating that it requires the interaction with HsbD. In contrast, deleting *hsbD* has no influence on the hyperbiofilm phenotype associated with the non-phosphorylable form of HsbA (HsbA_S56A_). These data thus confirm the functional and direct link between HsbD and HsbA-P.

### HsbD impacts *P*. *aeruginosa* swarming motility

**Fig 4:**
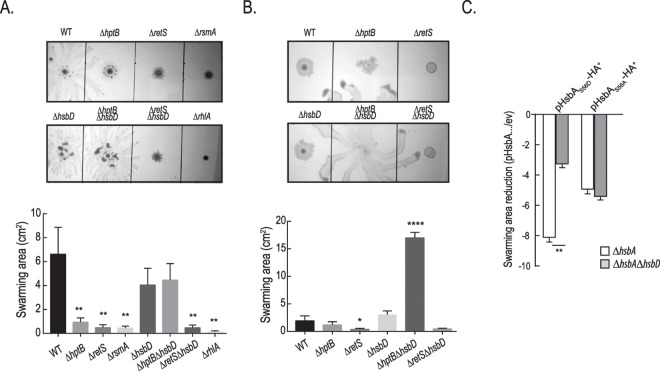
A Δ*hptBΔhsbD* mutant is hyper-swarming. Swarming motility of PAK wild type (WT) and PAKΔ*hptB*, Δ*retS*, Δ*rsmA*, Δ*hsbD*, Δ*hptB*Δ*hsbD*, Δ*retS*Δ*hsbD*, Δ*rhlA* mutant strains growing in minimal medium supplemented with glucose and casamino-acids **(A)** or nutrient agar plus glucose **(B)**. Surface area covered by the swarming cells (± standard deviation) was calculated by averaging data from four individual swarm plates (Student t-test, *, *p*< 0.01; **, *p* < 0.005; ****, *p* < 0.0001). **(C)** Fold reduction of swarming motility due to HsbA_S56D_ or HsbA_S56A_ overexpression relative to strains carrying the empty vector (ev) in Δ*hsbA* (white columns) and Δ*hsbA*Δ*hsbD* (grey columns) strains. Bacterial strains were grown in in minimal medium supplemented with glucose and casamino-acids.

It was previously reported that an *hsbA* mutant is hyper-swarming while an *hptB* mutant is not swarming [18]. We therefore evaluated the role of HsbD in this motility process (Fig 4). Swarming is an extremely variable phenomenon that depends on many factors including medium composition [34, 35]. Using minimal medium supplemented with glucose and casamino acids, we confirmed that deletion of *hptB*, *retS* or *rsmA* dramatically hampered swarming while the wild-type strain develops a large swarming area (Fig 4A) [20, 36]. In this condition, the deletion of *hsbD* does not affect overall swarming but changes its pattern with tendrils appearing shorter and tighter as compared to wild-type (Fig 4A). Deletion of *hsbA* results instead in a hyper-swarming phenotype that is still seen in the Δ*hsbA*Δ*hsbD* mutant (S5A Fig). When the swarming assay was performed on complex nutrient agar plates, the motility zone covered by the wild-type is severely reduced and likewise mutations in *hptB*, *hsbA* or *hsbD* did not impair swarming to the same extent as in minimal medium (Fig 4B and S5B Fig). However, when the *hsbD* mutation is introduced in the Δ*hptB* background the resulting strain displays a striking hyper-swarming phenotype (~9 fold increase in swarming area, Fig 4B). The fact that the *hsbD* deletion phenotype is visible only in the *hptB* mutant background suggests that HptB negatively controls HsbD directly and/or *via* HsbD-HsbA protein-protein interaction, as suggested by our previous data. To test whether the HptB regulation of HsbD was occurring via HsbD-HsbA protein-protein interaction, we tested the impact of HsbA_S56D_ or HsbA_S56A_ overproduction (Fig 4C). While overexpression of both HsbA variants was reducing swarming motility, as previously described [20], only the effect of HsbA_S56D_ (HsbA-P) was significantly attenuated in the strain deleted for *hsbD* (Δ*hsbA*Δ*hsbD*) compared to the Δ*hsbA* strain. These data confirm once again the functional link between HsbD and HsbA-P. However, the HsbA_S56D_ repression of swarming is not completely abolished in the Δ*hsbA*Δ*hsbD* mutant and this could be explained by previous observations showing that HsbA_S56D_ is potentially still able to interact with FlgM [20]. HsbD and HsbA regulation of swarming are therefore only partially overlapping and this is evident by the phenotype of the Δ*hptB*Δ*hsbA* mutant, which does not show hyper-swarming phenotype in rich medium (S5C Fig).

### HsbD modulates type IV pili assembly and twitching motility

**Fig 5:**
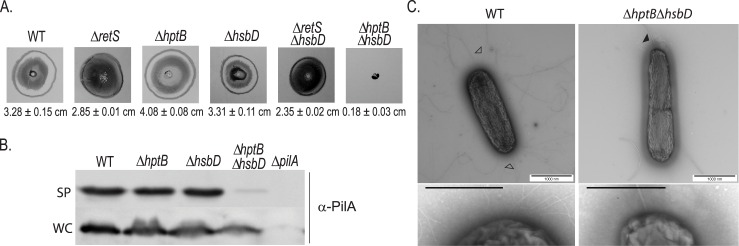
A Δ*hptBΔhsbD* mutant is impaired in twitching motility. **(A)** Twitching motility of PAK wild-type (WT) and Δ*retS*, Δ*hptB*, Δ*hsbD*, Δ*retS*Δ*hsbD*, Δ*hptB*Δ*hsbD* mutant strains. Twitching zones are visualized by crystal violet staining as indicated in Materials and Methods. At least three independent experiments were performed. Twitching diameters are indicated at the bottom of each panel ± standard deviation. **(B)** Analysis of PilA production and localization *via* Western blotting. Sheared-surface type IV pili (SP) and whole-cell extracts (WC) with identical samples were visualized *via* Western blotting. The following strains were analyzed: PAK wild type and PAKΔ*hptB*, Δ*hsbD*, Δ*hptB*Δ*hsbD*, Δ*pilA*. **(C)** TEM analysis of PAK wild-type (WT) and Δ*hptB*Δ*hsbD* mutant strains. Representative cells are shown entirely and insets illustrate higher magnification. Scale bar, 1000 nm and 200 nm, respectively. Closed arrowhead denotes flagellum, open arrowheads indicate representative type IV pili.

In many instances hyper-swarmers are poor twitchers [37–39] arguing that these two processes are inversely regulated. We thus tested HsbD impact on twitching motility (Fig 5). We showed that an *hptB* mutation indeed results in a slight increase in twitching (Fig 5A). Previous studies showed that overexpressing *hptB* results in decreased twitching, thus supporting the idea that HptB negatively influences this type of motility [27]. Whereas deleting the *hsbD* gene in the wild-type strain has no effect on twitching, this impact is most dramatic when the deletion is introduced in the Δ*hptB* background (Fig 5A). In this strain twitching motility is completely abrogated with the leading edge of the colony being identical to a *pilA* mutant as observed by light microscopy (S6 Fig). We further investigated the Δ*hptB*Δ*hsbD* mutant twitching defect by analyzing cell surface piliation (Fig 5B). Whereas the overall PilA production was not affected in the Δ*hptB*Δ*hsbD* mutant, only residual PilA protein was detected externally suggesting a coordinated role of HsbD and HptB in pili biogenesis/assembly. To validate this hypothesis, we directly monitored the presence of pili on the cell surface using transmission electron microscopy (TEM, Fig 5C). While wild-type cells possessed multiple polar pili, only the polar flagellum could be detected in the Δ*hptB*Δ*hsbD* mutant cells. Overall, our data suggest that HsbD positively influences twitching motility and in particular pili biogenesis/assembly. Since this effect is only observed in a mutant lacking HptB, we conclude that HptB has a negative impact on HsbD. The integration of HsbD in the HptB network again appears specific, as no variation in twitching motility is observed upon introduction of the *hsbD* mutation in a Δ*retS* background (Fig 5A) [36, 40]. We did not observe any significant effect of an *hsbA* deletion nor overexpression on twitching motility (S7 Fig). We therefore cannot exclude that HsbD could control twitching motility independently on HsbA. In agreement with this hypothesis, the Δ*hptB*Δ*hsbA* mutant is still twitching, with motility levels comparable to the Δ*hptB* mutant (S7B Fig). It is important to mention that the *fimV-like* gene (PA3340), previously associated with twitching, is found upstream of *hsbD* (Fig 1A) [29].

### HsbD is required for swimming motility and chemotaxis

**Fig 6:**
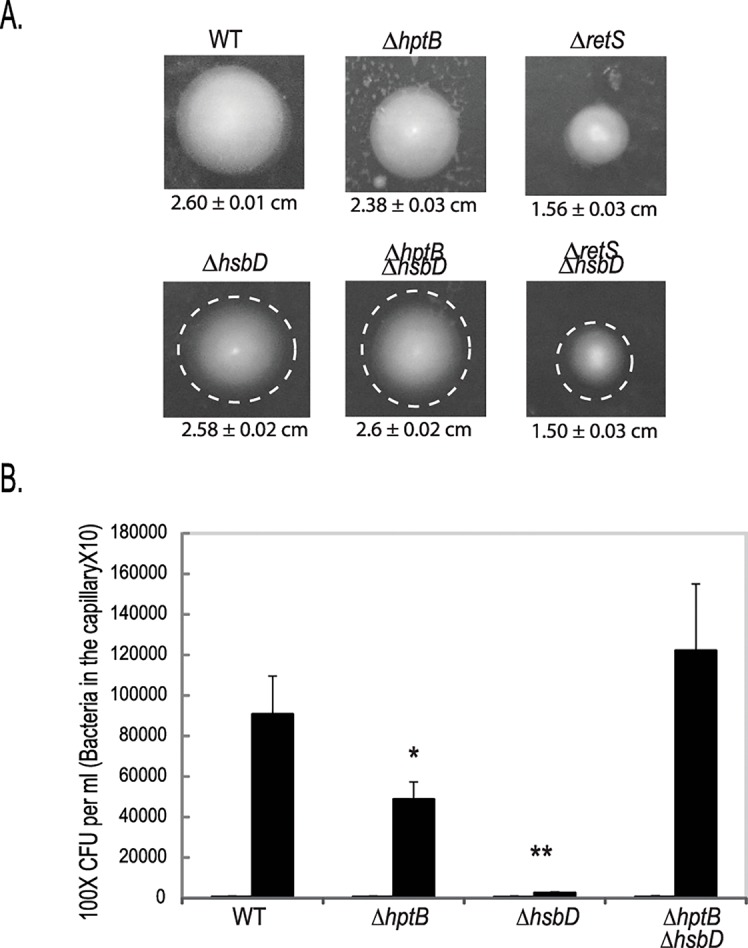
HsbD is regulating chemotaxis motility. **(A)** Swimming motility of PAK wild type (WT) and Δ*retS*, Δ*hptB*, Δ*hsbD*, Δ*retS*Δ*hsbD*, Δ*hptB*Δ*hsbD* mutant strains. White dashed circles correspond to the diameter of the WT strain. At least three independent experiments were performed. **(B)** Chemotactic response of PAK WT, *hptB*, *hsbD* and *hptB*/*hsbD* strains using 0.5% of casaminoacids as chemoatractant (black histograms) or PBS as control. The duration of chemotactic incubation time is 20 min. Each value is the average of three separate assay ± standard deviation (*, *p* < 0.05; **, *p* < 0.01).

*P*. *aeruginosa* possesses three main types of motility: swarming, twitching and swimming. Swimming motility is dependent on flagella and relies on a chemotactic response. It has been previously proposed that HptB signaling can influence chemotaxis [27]. Here, we confirm that an *hptB* mutant is slightly impaired in swimming as is a *retS* mutant (Fig 6A) [36]. Interestingly, the swimming area of a Δ*hsbD* strain remains approximately identical to the wild-type strain but the outer swim ring appears less dense (Fig 6A). This suggests that the impairment associated with the *hsbD* mutation is due to a chemotactic defect. A chemotaxis experiment was thus performed using a solution of casaminoacids (0.5%) as chemoattractant (Fig 6B). We confirmed a decrease in the chemotaxis response of the *hptB* mutant [27] and we observed a full chemotactic deficiency of the Δ*hsbD* strain. Interestingly, chemotaxis is restored to wild-type levels in the Δ*hptB*Δ*hsbD* mutant, further highlighting the antagonism between HptB and HsbD. The extent of this chemotaxis behavior will need to be further characterized. However, upstream of *hsbA* two genes involved in chemotaxis can be found (Fig 1A). PA3348 encodes a putative methyltransferase (CheR-like) whereas PA3349 encodes a CheW-like adaptor molecule. Further upstream is a cluster of four genes including, PA3350 (*flgA*-like), *flgM*, PA3352 (*flgN*-like) and PA3353 (Fig 1A). The DGC activity of HsbD might thus influence flagellar function by modulating the activity of some of these gene products. Notably the PA3353 PilZ domain-containing protein was previously proposed to bind c-di-GMP and shown to affect swimming motility in *P*. *putida* and swarming motility in *P*. *aeruginosa* [41–45]. Overall our data suggest that HsbD activity might not necessarily impact swimming motility *per se* but could significantly influence the flagellar based movement both on liquid and on surface.

### SadC and HsbD differentially impact HptB-dependent phenotypes

**Fig 7:**
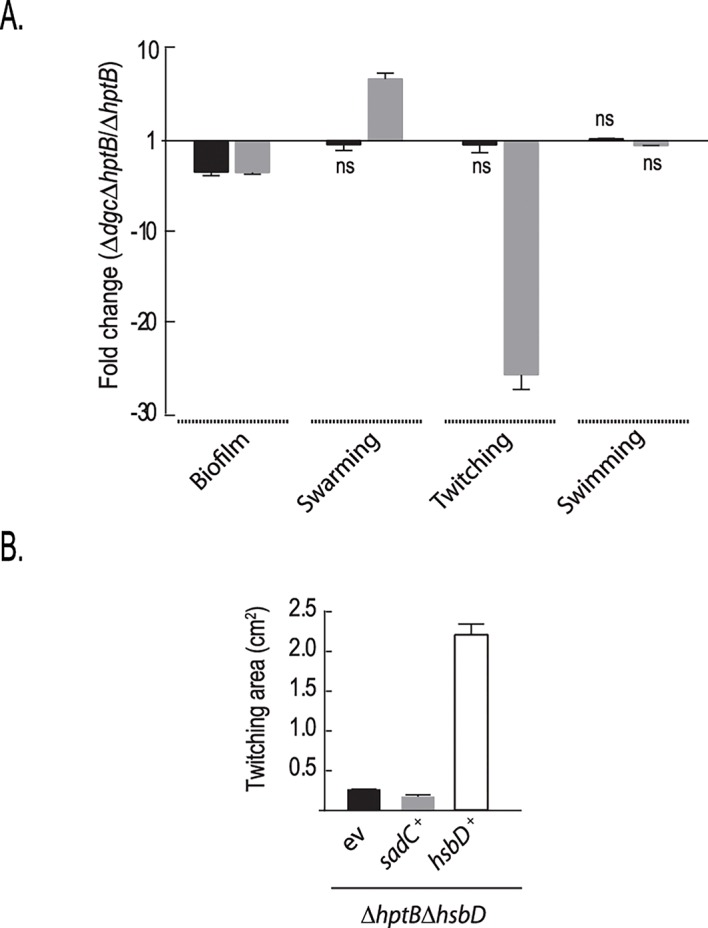
Role of HsbD and SadC in the HptB pathway. **(A)** Relative fold change difference in biofilm formation, swarming, twitching and swimming motilities of either the PAKΔ*hptB*Δ*sadC* (black) or Δ*hptB*Δ*hsbD* (grey) mutant compared to the Δ*hptB* mutant strain (ns: not significant). **(B)** Twitching motility of PAK Δ*hptB* and Δ*hptB*Δ*hsbD* mutant strains carrying either a pMMBRMCS4 empty plasmid (-) or the pBBR3347 plasmid overexpressing *sadC* (+). Each value is the average of three different cultures ± standard deviation (Student t-test, **, *p* < 0.01).

The hyper-biofilm phenotype of an *hptB* mutant was reduced to wild-type levels when deleting either *sadC* or *hsbD* in this background ([25] and Fig 2). These findings suggested that both DGCs might respond to input from the HptB pathway. We thus asked whether the Δ*sadC*Δ*hptB* and the Δ*hsbD*Δ*hptB* mutants display similar phenotypes, in other words if the SadC and HsbD regulatory effects are redundant or not. We compared the biofilm, swarming, twitching and swimming phenotypes of the double mutants and the Δ*hptB* parental strain (Fig 7A). We found that deletion of *sadC* in a Δ*hptB* background affects biofilm formation in a manner similar to *hsbD* deletion but, in contrast with the *hsbD* deletion, none of the motility behaviors. To confirm this observation, we introduced the *sadC* gene *in trans* (pBBRMCS4-*sadC*) in the Δ*hptB*Δ*hsbD* mutant (Fig 7B). The resulting recombinant strain overexpressing *sadC* is still impaired in twitching motility, a phenotype which could be readily complemented upon introduction of *hsbD in trans*. Altogether, these observations further support the idea that diguanylate cyclases can adopt specific roles in the control of a bacterial lifestyle, particularly in respect to motility.

### HsbD localizes at the cell pole

**Fig 8:**
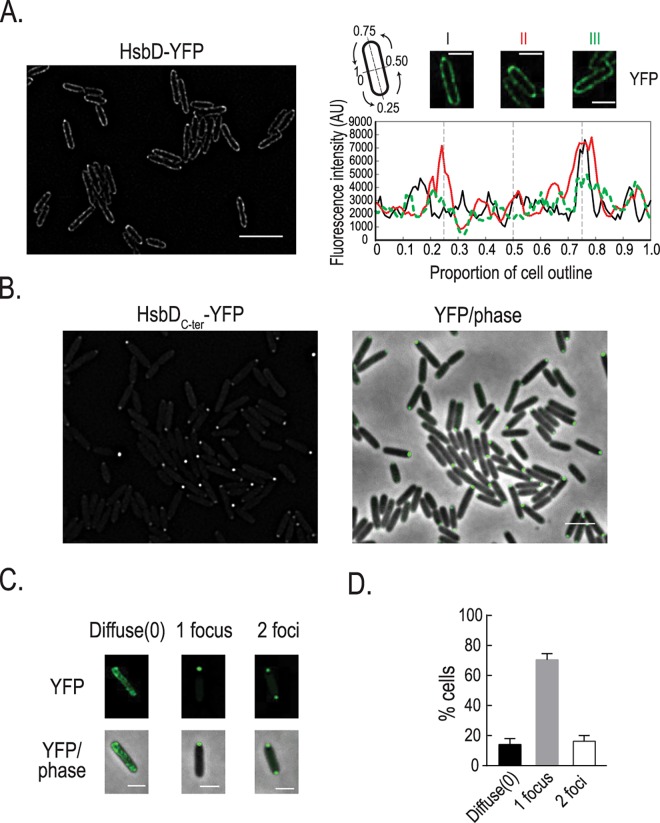
HsbD polar localization in *P*. *aeruginosa*. **(A)** Localization of HsbD-YFP (green) in cells grown in LB medium and induced with 100μM IPTG for two hours (left panel). Three representative cells (right panel) where chosen for the quantification of the fluorescence intensity across the cell contour. Black line: cell I, red: cell II and green: cell III. A pseudo-colored fluorescence image (green YFP) of each cell is shown on top of the graph. Cartoon: representation of coordinates reported in the graph. Scale bar = 3 μm **(B)** Localization of the GGDEF-containing domain of HsbD (HsbD_C-ter_-YFP) in cells grown in LB medium and induced with 100μM IPTG for two hours. First column shows fluorescence images (YFP) while the second column the overlay of the fluorescence channel (in green) and the phase contrast image. Scale bar = 2 μm. **(C)** Representative cells where chosen to illustrate the three localization patterns of HsbD_C-ter_-YFP. Scale bar = 1 μm **(D)** Quantification of the distinct HsbD_C-ter_-YFP localization patterns. Error bars represent the standard deviation (n = 3 replicates of more than 200 cells each). At least three independent experiments were performed.

In *P*. *aeruginosa*, as in many rod shape bacteria, motility machineries like flagellum or type IV pili are located at the poles. Having demonstrated the importance of HsbD for *P*. *aeruginosa* motility, we examined its cellular localization (Fig 8). In order to visualize HsbD in cells we fused the full-length protein and the DGC domain to the yellow fluorescent protein (*venus*, *i*.*e*. *yfp* derivative) generating HsbD-YFP and HsbD_C-ter_-YFP chimera, respectively. Functionality of the tagged full length protein and the truncated GGDEF domain was verified by monitoring Congo-Red staining of *E*. *coli* strains carrying these constructs and by complementation of the *P*. *aeruginosa* Δ*hptB*Δ*hsbD* mutant (S8 Fig). We subsequently induced expression of the YFP-tagged HsbD constructs for 2 hours in cells grown in LB. Analysis of HsbD-YFP shows that HsbD localizes to the cell periphery with some local concentration at the cell pole(s) (Fig 8A). Polar localization of the HsbD_C-ter_-YFP chimera is even more obvious, thus suggesting that the transmembrane domains of HsbD might have a role in controlling its polar localization (Fig 8B). Overall, the cell population appears to have three distinct localization patterns, with 86% of cells displaying HsbD_C-ter_-YFP at either one or both poles, and a small fraction (14%) having a rather diffuse fluorescence (Fig 8C and 8D).

### HsbD and FlhF colocalize transiently at the pole

**Fig 9:**
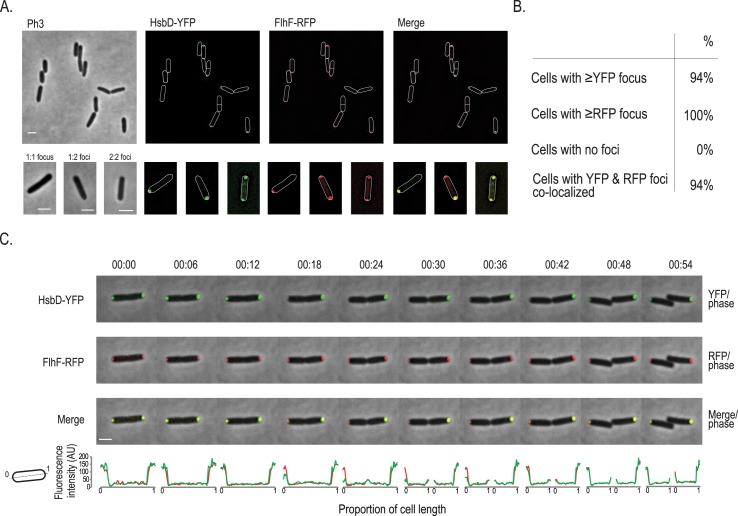
Dynamics of HsbD and FhlF cellular (co-)localization. **(A)** Localization of HsbD-YFP (green) and FhlF-RFP (red) in a *P*. *aeruginosa* wild-type strain. Scale bar = 2 μm. Bottom panel: three representative patterns (1:1, 1:2 foci and 2:2 YFP:RFP foci) are illustrated in a closer view. Scale bar = 1 μm. Cell boundaries are delineated in white. **(B)** HsbD and FhlF polar localization frequency and their colocalization. More than 200 cells were analyzed from different fields. At least three independent experiments were performed. **(C)** Dynamics of HsbD-YFP (green) and FhlF-RFP (red). An overlay of the fluorescence channel(s) and the phase contrast image illustrate HsbD-YFP and FlhF-RFP subcellular localization pattern. Fluorescence images are shown in S6 Fig. Scale bar = 1 μm. Bottom panel shows the quantification of the HsbD-YFP (green) and FhlF-RFP (red) fluorescence intensity across the cell length. Cartoon: representation of coordinates reported in the graph.

The flagellar GTP-binding protein FhlF has been shown previously to be polarly localized and to control localization of the flagellum in *P*. *aeruginosa* [46, 47]. As we observed that HsbD has an impact on motility, including flagellum-based swarming and swimming, we performed co-localization studies of YFP-tagged HsbD and RFP-tagged FlhF co-expressed in wild-type cells (Fig 9). We found that the two proteins consistently co-localize at the cell pole, although not in all cases. This is due to a different localization behavior of the proteins, since FlhF is primarily bipolar and HsbD can be present at either one or both cell poles (Fig 8C). Therefore three different situations could be identified (Fig 9A, lower panel): (i) HsbD and FlhF colocalize at one pole (1:1 focus), (ii) at both poles (2:2 foci), (iii) HsbD is located at one pole while FlhF is at both poles, resulting in YFP:RFP merging only for one out of two RFP foci (1:2 foci). We then performed time-lapse experiments to follow the localization dynamics of HsbD and its colocalization with FlhF (Fig 9C and S9A Fig and S1 and S2 Movies). FlhF position during cell division has been previously described to be coordinated throughout the cell cycle [48]. Briefly, FlhF localizes at the old cell pole and when the cell engages in division it is recruited to the opposite new pole (emerging from the previous cell division), where a new flagellum will be subsequently assembled [48, 49]. Newly-formed puncta can thus be seen at the new pole during cell division, reestablishing a FlhF bipolar localization pattern in daughter cells with the brightest intensity located at the flagellated pole [48]. We observed the same “trends” for FlhF localization (Fig 9C, middle panel) which are bipolar localization of FlhF upon completion of cell division at time 54 min with brightest intensity at the old pole and weaker intensity at the new pole. In this exact same setting (Fig 9C, upper panel), HsbD colocalizes with FlhF to the poles when cells initiate division. However, upon completion of septation HsbD transiently disappears from one of the poles (18–30 min) and reappears at the “old pole” of the newly formed daughter cell after division is completed (36–54 min). Following cell division over a second generation, we observed that the pole where HsbD transiently disappear is the pole emerging from the most recent cell division event, indicating HsbD permanently “sits” at the pole with the inherited flagellum (S9B Fig). Overall, our data show a clear asymmetric and dynamic distribution of HsbD during cell division, suggesting that the signaling network differentially impacts on the motility behavior of both daughter cells. Furthermore, dynamic localization of HsbD explains the occurrence of the three HsbD-YFP patterns (Fig 8) and the “transient” HsbD colocalization with FlhF (Fig 9A and 9B).

### HptB affects the overall distribution of HsbD localization

**Fig 10:**
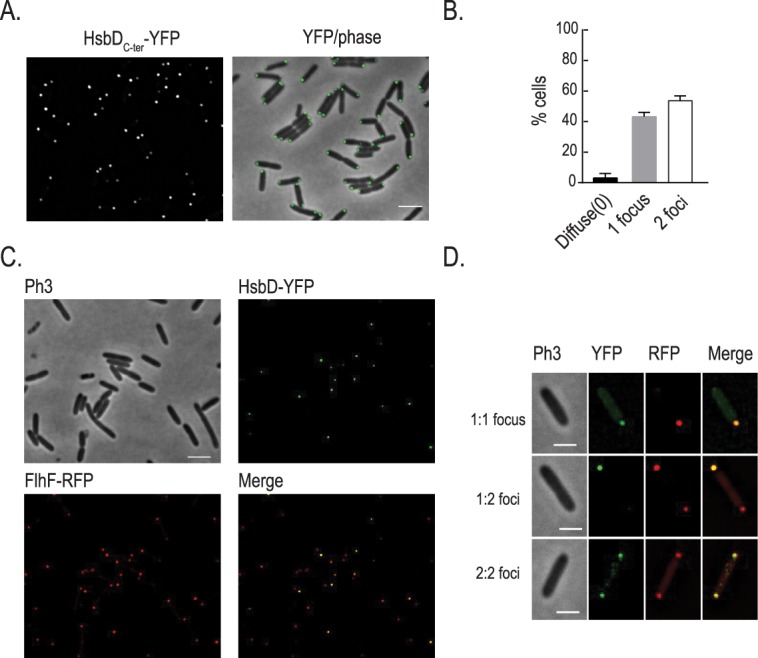
HsbD polar localization in a *P*. *aeruginosa* Δ*hptB* mutant. **(A)** Localization of the GGEEF-containing domain of HsbD (HsbD_C-ter_-YFP) in cells grown in LB medium and induced with 100μM IPTG for two hours. Scale bar = 2 μm. **(B)** Quantification of the distinct HsbD_C-ter_ localization patterns in cell population. Error bars represent the standard deviation (n = 3 replicates of more than 200 cells each). **(C)** Localization of HsbD_C-ter_-YFP (green) and FhlF-RFP (red) in a Δ*hptB* mutant strain. Scale bar = 2 μm **(D)** Three representative patterns are illustrated in a closer view. Scale bar = 1 μm.

Because we showed a functional link between HsbD and the HptB pathway, we assessed a possible influence of HptB on HsbD localization. To address this question, YFP-tagged HsbD was visualized by fluorescence microscopy in a *P*. *aeruginosa* Δ*hptB* mutant strain (Fig 10). Overall, the *hptB* mutant cells exhibited the same HsbD polar localization as observed in wild type (Fig 10A and 10B as compared to Fig 8B and 8D). However, the frequency of cells exhibiting HsbD_C-ter_-YFP at both cell poles was significantly increased (15% in wild type *vs*. 51% in the Δ*hptB* mutant). Yet, when colocalization with FhlF was tested, we observed the same three distribution patterns as in wild-type cells (Fig 10C and 10D). These results thus suggest that HptB interferes with polar localization of HsbD, while it does not seem to affect the dynamic behavior of HsbD during the cell cycle. Given that c-di-GMP levels are increased in the Δ*hptB* mutant in an HsbD-dependent manner (Fig 2A), it is possible that dynamic localization and activity of HsbD are in fact coupled. If so, the dynamic behavior of HsbD might be key to understanding how this DGC contributes to different cellular processes during surface adaptation of *P*. *aeruginosa*.

## Discussion

The regulatory networks that switch *P*. *aeruginosa* from a planktonic to a biofilm lifestyle are central to the fine-tuning of bacterial adaptation and the success of host colonization. These have been described in previous studies and involve complex pathways, such as the RetS and HptB signaling cascades, which feed into the Gac/Rsm regulatory module [50].

### The HsbA anti-anti-sigma factor is a relay in the HptB pathway

HptB, like other proteins regulating RsmY/RsmZ sRNA levels (*e*.*g*. LadS, GacAS, SagS), has been proposed to be important for the initial interaction of planktonic cells with surface and subsequent biofilm development [22, 26]. When compared to wild type, the *hptB* mutant produces more exopolysaccharides [17], attaches more easily to surface, increases twitching motility while it decreases swimming and swarming movements (Figs 2 and 4–6). In other words, HptB inactivation switches the bacterial lifestyle towards a biofilm state. The HptB pathway consists of a multi-step phosphorelay cascade and it relies on the so-called HsbA partner-switching mechanism, wherein the interacting partners of the HsbA anti-anti-sigma factor are the FlgM anti-sigma factor or the HsbR response regulator [20]. The switch of interaction between either one of these partners depends on the phosphorylation status of HsbA: FlgM when HsbA is dephosphorylated or HsbR if HsbA is phosphorylated. In this model, dephosphorylated HsbA would promote FliA(σ^28^)-dependent transcription of class IV flagellar genes, hence swimming and swarming motilities [51–53]. Phosphorylated HsbA would instead repress swarming motility and intersect with the Gac/Rsm cascade for the control of biofilm formation [17, 20].

### The diguanylate cyclase HsbD is central in the HptB pathway

**Fig 11:**
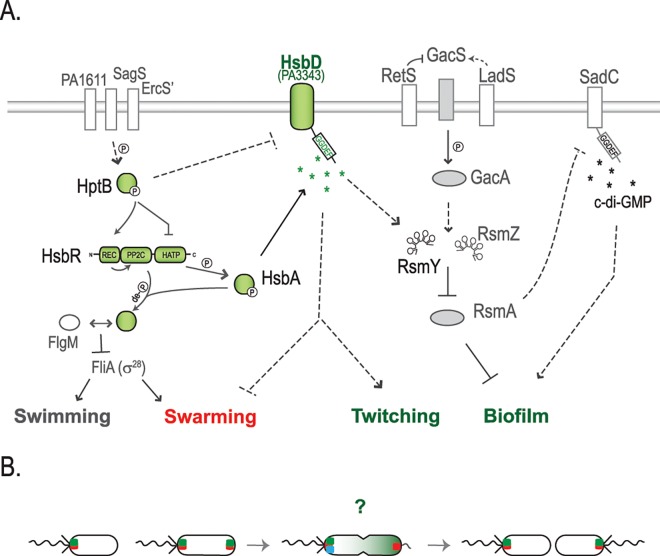
Working model for the HptB signaling pathway in *P*. *aeruginosa*. **(A)** HptB is activated (*i*.*e*. phosphorylated) via either one of three orphan sensor kinase hybrids: PA1611, ErcS’ (PA1976) or SagS (PA2824) [26–28]. When phosphorylated, HptB transfers a phosphoryl group to the HsbR receiver domain (HsbR-P), thus repressing HsbR kinase activity while activating its phosphatase activity. HsbR-P dephosphorylates the anti-anti-sigma factor HsbA. Dephosphorylated HsbA leads to dissociation of the HsbA-HsbR complex and a subsequent stable sequestration of the anti-sigma factor FlgM by HsbA [20]. The interaction HsbA-FlgM induces flagellar genes expression, by allowing the release of the FliA sigma factor (σ^28^) [51]. When HptB is inactive (dephosphorylated or in an *hptB* mutant), swimming and swarming are no longer supported. Instead, the HsbR kinase phosphorylates HsbA (HsbA-P) and HsbA-P interaction with HsbD leads to an increase of c-di-GMP and RsmY levels. The activation of HsbD strengthens swarming repression and results in hyper-biofilm and hyper-twitching phenotypes. This cascade of events is in agreement with the waves of regulatory actions that cause bacteria progression from an early surface attachment and colonization (swimming and swarming) to the development of mature sessile biofilms [15, 26, 56]. The symbol ➔ indicates positive regulation, while -¦ repression and de–P (de)phosphorylation. Dashed lines suggest a probable indirect regulation. Components of the HptB signaling pathway are colored in green. **(B)** Localization dynamics of HsbD (green square) during cell division. FhlF (red square) dynamics is reported as commented previously by Burrows LL [49]. Green gradient in the cell undergoing division illustrates bimodal distribution of c-di-GMP levels as reported by Christen and colleagues [14].? Question mark represents possible scenarios on the asymmetric partitioning of HsbD during cell division in respect to flagellum biogenesis and DipA (blue square) localization. For details see text.

Here, we found that the HptB pathway requires the diguanylate cyclase HsbD (PA3343) to fulfill its regulatory action. HsbD was previously proposed to be an active DGC in a systematic screen correlating DGCs and PDEs activities to *P*. *aeruginosa* phenotypic outputs, *i*.*e*. biofilm and cytotoxicity [16]. In the current study, we show that HsbD binds c-di-GMP, *via* its I-site, and we propose that this DGC has a specific link with the HptB pathway as shown in the model presented in Fig 11. This model integrates our new data and depicts the regulation of biofilm formation and motility *via* the HtpB cascade. HsbD is central for the HptB-regulated lifestyle switch, since in the Δ*hsbD*Δ*hptB* mutant, biofilm, c-di-GMP and RsmY levels are reset to a wild-type condition (Fig 2). The HsbD connection with the HptB pathway is further revealed with the observed direct interaction between the DGC and the phosphorylated form of HsbA (HsbA-P). The functional link between these two partners is validated by a nearly identical biofilm phenotype for the Δ*hptB*Δ*hsbD* and Δ*hptB*Δ*hsbA* mutants. Besides, the deletion of *hsbD* is abolishing the increase of biofilm dependent on HsbA-P overexpression (Fig 3C). Finally, there is no evidence of HsbD being phosphorylated by HsbA-P, but HsbD does not contain a receiver domain, unlike the well-characterized DGCs WspR or PleD [54, 55].

### The HptB cascade includes multiple paths to control motility

From the above we concluded that HsbD relies on HsbA for biofilm formation, however we also provide data indicating that such connection is partially required for swarming and not at all for twitching. Indeed, we observed that deletion of *hsbD* or *hsbA* in a *hptB* mutant background does not result in strictly identical motility behaviors. In particular, the Δ*hptB*Δ*hsbD* mutant, despite forming wild-type levels of biofilm, does not assemble surface pili and displays more intense swarming (Figs 2, 4 and 5), while the Δ*hptB*Δ*hsbA* mutant displays twitching and swarming levels similar to the Δ*hptB* mutant. This is suggesting that in the absence of HptB, HsbD might repress swarming and induce twitching independently of HsbA. However, this conclusion should be mitigated since we observed that overexpressing HsbA-P strongly represses hyper-swarming of the Δ*hsbA* mutant, while such repression is weaker in a Δ*hsbA*Δ*hsbD* mutant (Fig 4C), thus suggesting that swarming regulation by the HptB cascade involves both HsbD and HsbA, and at least in part their interaction. Concerning twitching, our data show functional connections between HptB and HsbD that do not involve HsbA-P, which implies the existence of non-linear pathways leading to HsbD action.

Overall, the sequence of events controlled by the HptB/HsbA/HsbD pathway thus reveals an appealing scenario with respect to the coordination of motility during biofilm development [15, 56][15, 56]. When HptB is phosphorylated, HsbA would promote FliA action and favor planktonic growth (swimming), swarming and initial surface attachment [57]. Once HptB control is relieved (HptB unphosphorylated or deleted), FlgM would sequester FliA, while HsbA-P would rely on HsbD to allow the progression in the biofilm cycle, by further repressing swarming, reinforcing twitching motility and biofilm maturation.

### HsbD and c-di-GMP signaling

The global levels of c-di-GMP in the single *hsbD* mutant are comparable to the wild-type, yet the strain displays a mild swarming alteration and it seems to be affected in chemotaxis (Figs 4 and 6). Additional lines of evidence exist for a link between c-di-GMP and chemotaxis control. In *P*. *aeruginosa*, the DGC WspR is associated with a chemotactic system responding to growth on surfaces [58], while in *Azospirillum* a chemotactic receptor that potentiates bacterial motility upon intracellular increase of c-di-GMP has been described [59]. One possible explanation for the discrepancy between c-di-GMP levels and the Δ*hsbD* phenotypes is that, in presence of HptB (inactive or HptB-P), HsbD could affect only a subcellular c-di-GMP pool while not disturbing the global c-di-GMP levels. Our observation that HsbD localized at the pole and that HptB may inhibit this localization is in agreement with this hypothesis (Figs 8 and 10). The idea of c-di-GMP discrete pools was previously proposed in *P*. *aeruginosa* for SadC and RoeA DGCs, where no correlation was observed between global c-di-GMP levels and the phenotypic output of *sadC* and *roeA* deletions [60]. Another example of subcellular localization of a DGC at the cell pole is PleD from *Caulobacter crescentus*, where the protein is activated by phosphorylation during the cell cycle, and as a consequence relocates to the pole [61, 62]. It was suggested that localization of active PleD to the old cell pole contributes to a spatial gradient of c-di-GMP in dividing *Caulobacter* cells [63]. Here we showed that HsbD dynamically localizes to the *P*. *aeruginosa* cell poles during the cell cycle. When *P*. *aeruginosa* divides, one daughter cell inherits the flagellum located at the old pole while the other progeny rapidly assembles a new one [64]. Recently it was shown that the daughter cell inheriting the “old flagellum” has reduced intracellular c-di-GMP levels as compared to its sibling [14]. Interestingly, bimodal distribution of c-di-GMP during cell division relies on the PDE Pch (PA5017, named also DipA) and on the asymmetrically positioned chemotaxis machinery (*i*.*e*. CheA) [65]. Our data indicate that polar localization of HsbD is transiently and asymmetrically abandoned during cell division in one of the two daughter cells (Fig 9) emphasizing a clear asymmetry in HsbD distribution at this specific stage of the cell cycle. We also present indirect evidence suggesting that HsbD and DipA should both co-localize at the pole with the inherited flagellum (S9 Fig). It is not clear how the dynamic changes of HsbD localization relate to the observed asymmetric activity of Pch(DipA) during division but colocalization of HsbD with the flagellar system, suggests that at least temporarily the two *P*. *aeruginosa* daughter cells may have different motility behaviors and that HsbD is involved in this control (Fig 11B).

### HsbD specificity

The in-depth characterization of the HptB/HsbD system presented in this study has proven to be a good example to tackle the yet unresolved matter of c-di-GMP signaling specificity. Indeed, one of the major unresolved or poorly documented questions is how DGCs can stimulate specific cellular processes by producing a signal, c-di-GMP, that is freely diffusible and that can potentially affect multiple regulatory pathways and phenotypic outputs simultaneously? Here, we present observations that partly explain how c-di-GMP signaling reaches specificity. Firstly, we show a tight link between the HsbD diguanylate cyclase and the HptB pathway. Secondly, we propose a local action of HsbD at the cell pole. Thirdly, we present evidence on how two DGCs, *i*.*e*. HsbD and SadC, are intersecting within the same global regulatory network (Gac/Rsm) in a hierarchical order. The Gac/Rsm cascade contributes to the high c-di-GMP regime necessary for *P*. *aeruginosa* biofilm formation. Recently, we showed that SadC, a Gac/Rsm controlled DGC, is central for the regulation of biofilm formation. Indeed, the high level c-di-GMP regime and associated hyper-biofilm observed in *retS*, *rsmA* or *hptB* mutants, and in a *ladS* overexpression strain, was reversed to wild-type levels by deleting the *sadC* gene [25]. Here we show that the *hsbD* mutation restores wild-type c-di-GMP levels and behavior from a hyper-biofilm regime in the *hptB* mutant, but not in the *retS* mutant. Furthermore, although HsbD and SadC both contribute to the HptB dependent regulation of biofilm formation, they have a different impact on the HptB-mediated motility (Fig 7A). We therefore suggest that HsbD and SadC act at distinct position in the c-di-GMP network that controls *P*. *aeruginosa* behavior (Fig 11A). Indeed, a *sadC* mutant is defective in biofilm formation and hyper-swarming, while no defect in swimming or twitching is observed [60, 66]. Instead, an *hsbD* mutant has a weak alteration of its swimming pattern, but displays a chemotactic defect. We propose that SadC acts as a central DGC for the entire Gac/Rsm cascade and we place it downstream of RsmA, since RsmA directly represses SadC production [25]. Instead, HsbD acts as a DGC specifically associated with the HptB pathway as it exclusively regulates RsmY and not RsmZ levels (S3 Fig). The role of HsbD is thus upstream of RsmA, and consequently of SadC. The fact that SadC cannot complement for the lack of HsbD in the *hptB* mutant background is in support of such a model (Fig 7B).

The acquisition of *hsbD* in *P*. *aeruginosa* and the genetic reorganization that brought together *hsbD*, *hptB/hsbA/hsbR* and additional flagellar and chemotactic genes to form a novel entity involved in the control of motility and biofilm reveals an interesting scenario on the possible evolution of c-di-GMP signaling. Indeed, the genomic reorganization might have led to the specific interaction between HsbD and the HptB pathway. To support this idea, it would be necessary to study HsbD (role and connection with HptB) on those few non-*aeruginosa* strains that acquired *hsbD* but in which the flagellar genes reorganization did not occur (*e*.*g*. *P*. *mendocina*, Fig 1).

In conclusion, our study on the HsbD diguanylate cyclase highlights new strategies by which c-di-GMP could specifically influence a bacterial lifestyle and enlarge the category of proteins associated with c-di-GMP signaling, as shown here with the anti-anti-sigma factor HsbA. It further provides evidence on how a complex regulatory network gives c-di-GMP signaling output specificity by a tight control of its synthesis and local action.

## Materials and Method

### Strains and plasmids

Bacterial strains and plasmids used in this study are listed in S1 and S2 Tables, respectively. Cells were grown in Luria Broth (LB) [67, 68] in 100 ml Erlenmeyer flasks filled with 20 ml of medium, with shaking at 180 rpm and at 37°C. LB agar (NA) was used as a solid medium. Congo red staining assay was performed at 30°C on tryptone (10 g/l) agar (1%) plates supplemented with 40 μg/ml Congo red and 20 μg/ml Coomassie brilliant blue. When required, antibiotics were added to these media at the following concentrations: 100 μg/ml ampicillin, 25 μg/ml tetracycline and 10 μg/ml gentamicin for *E*. *coli*; and 300 μg/ml carbenicillin, 50 μg/ml gentamicin and 125 μg/ml tetracycline, 2000 μg/ml streptomycin for *P*. *aeruginosa*.

### Phylogenetic analysis

The phylogenetic analysis for 48 *Pseudomonas* species carrying an *hptB* orthologue was conducted by concatenated analysis considering the *gyrA* and *gyrB* genes. Sequences were retrieved from the NCBI RefSeq database [69] and aligned with MUSCLE [70]. Sequences of *Xanthomonas campestris pv*. *campestris* were added as outgroup. All positions with less than 95% site coverage were eliminated. That is, fewer than 5% alignment gaps, missing data, and ambiguous bases were allowed at any position. There were a total of 1481 positions in the final dataset. The evolutionary history was inferred by constructing a phylogenetic tree using the Neighbor-Joining method in MEGA6 [71, 72]. The evolutionary distances were computed using the Poisson correction method [73] and are in the units of the number of amino acid substitutions per site.

### Cloning procedures

DNA cloning and plasmid preparation were performed according to standard methods [74]. PCR primers were designed with restriction sites at their ends for subsequent digestion and ligation into the specific vector. Restriction and DNA-modifying enzymes were used following the instructions of the manufacturers. Transformation of *E*. *coli* DH5α, *E*. *coli* TOP10 (for cloning) and *P*. *aeruginosa* was carried out by electroporation [75]. All plasmids were verified by sequencing. Site-directed mutagenesis was performed using the QuikChange II site-directed mutagenesis kit according to the manufacturer's instructions (Agilent Technologies). All mutations were confirmed by DNA sequencing.

### Gene replacement mutants

For the inactivation of the PA3343 (*hsbD*) gene in the *P*. *aeruginosa* PAK chromosome previously described protocol was followed [25]. Briefly, a 504-bp fragment containing the upstream region and the first 2 codons of PA3343 and a 501-bp fragment containing the *hsbD* stop codon were amplified by PCR using the primer couples pPA3343.1/ pPA3343.2 and pPA3343.3/ pPA3343.4, respectively. Mutator fragments were constructed by PCR amplification of upstream and downstream fragments using the primer couple pPA3343.1/ pPA3343.4 and cloned into pKNG101. Plasmid pKNG101Δ*PA3343*, carried by *E*. *coli* TOP10, was then introduced into *P*. *aeruginosa* PAK by triparental mating, using the helper strain *E*.*coli* HB101 (pRK2013). Transconjugants were isolated on Pseudomonas Isolation Agar (Difco) supplemented with appropriate antibiotics. Deletion mutants were selected in 5% sucrose after 2 days of incubation at room temperature. Deletions were confirmed by sequencing using external primers pPA3343.5/pPA3343.6. The resulting strain PAKΔ*hsbD*, carried an in-frame Δ*hsbD* mutation. Double mutants were obtained by crossing plasmid pKNG101Δ*PA3343* into PAKΔ*hptB*, PAKΔ*retS* and PAKΔ*hsbA* as described above, giving strains PAKΔ*hptB*Δ*hsbD*, PAKΔ*retS*Δ*hsbD* and PAKΔ*hsbA*Δ*hsbD* respectively. In all mutants described here, the deletions were confirmed by PCR. The *hsbD* deletion was complemented with fragments carrying *hsbD* gene. The fragments had been amplified by PCR with primers pPA3343ov.1F- pPA3343ov.2R and cloned into pBBRMCS-4.

### β-Galactosidase assays

β-Galactosidase experiments were performed as described previously [68], with P. aeruginosa strains grown in LB medium. Data are mean values of three independent samples ± standard deviations.

### Biofilm assays

Quantification of biofilm formation was performed in 24-well polystyrene microtiter plates as previously described [25]. The plates were incubated for 10 h at 37°C and biofilms were stained with 0.1% crystal violet solution. The dye bound, which is proportional to the biofilm produced, was solubilized with 96% (v/v) ethanol and the absorption was photometrically measured at 600 nm (OD_600_). Data are mean values of three independent samples ± standard deviations.

### Motility assays

Motility assays were carried out essentially as previously described [76, 77]. Swim assays were carried out on 10 g/L tryptone, 5 g/L NaCl, 0.3% agar (Merck) plates. 0.5 μl of standardized overnight culture was injected below the surface of the agar and plates were incubated at 30°C overnight. Twitch assays were carried out on 1% LB agar plates and bacteria were inoculated by picking a colony using a sterile tips and stabbing to the bottom of the plates, which were incubated at 37°C for two days. The agar was then peeled off the plate, and cells were stained with crystal violet for visualization. Swarming assays were performed in plates consisted of 0.5% (wt/vol) Difco bacto-agar with either 8 g/liter Difco nutrient broth and 5 g/liter glucose or with MMP MMP medium supplemented with 20 mM glucose and 0.1% (w/v) casamino acids [76, 78]. Cells were inoculated onto swarm plates using 5μl of standardized overnight cultures and incubated overnight at 37°C. Pictures are taken from a representative plate out of five independent experiments. ImageJ software (NIH) was used to determine the area of the plate surface covered by the bacteria as previously described [79].

### PilA immunoblotting

Detection of sheared surface type IV pili and cell-associated pilin was performed as previously described [80]. For each strain, cells were harvested from confluent lawn LB agar plates grown at 37°C for 20 hours. Every sample was normalized to an O.D. 1.0 before loading it onto a 15% SDS-polyacrylamide gels. The gel was migrated and transferred to a nitrocellulose membrane at 3 mA/cm2. After transfer, membranes were blocked overnight in blocking buffer (5% milk powder, 0.1% Tween 20 in Tris-buffered saline, pH 8.0). A 1:5000 dilution of primary anti-PilA antibody was used. Secondary antibodies conjugated to horseradish peroxidase were used at a dilution of 1:5000. Western blots were developed using Super-Signal West Pico Chemiluminescent Substrate (Pierce) and visualized on a LAS3000 Fuji Imager.

### Chemotaxis Assays

Chemotaxis assays were performed as previously described [81]. Briefly, 100 μl (OD_600_ 0.2) of a bacterial suspension grown to stationary phase and 100 μl of a casamino acids solution (0.5%) were used. Incubation time and temperature (20 min) were optimized. Dilutions were plated onto LB plates and triplicate plate count of CFU was performed for each assay.

### Measurement of c-di-GMP levels

Intracellular c-di-GMP levels were estimated by the use of the *cdrA-gfp* reporter fusion, as previously described [25]. Experiments were done in triplicate and data are presented as relative fluorescent units (RFU), which are arbitrary fluorescent units, corrected for cell density, ± standard deviations. Alternatively, c-di-GMP levels were quantified by liquid-chromatography mass spectrometry (LC-MS/MS). Strains were grown to stationary phase in 50 ml of LB medium and c-di-GMP was extracted as described previously [82]. LC-MS/MS analysis was performed at the BIOLOG Life Science Institute (Biolog, Bremen). Samples of interest were compared to a standard curve derived from measurements of known concentrations of pure c-di-GMP to determine the concentration (in nM) of c-di-GMP in the samples. The data were then normalized to the total protein content of the sample determined by Bradford assay. For each strain, experiment was done in biological triplicate and LC-MS/MS measurements were repeated in duplicate. Data are presented as pmol of c-di-GMP/mg of total protein.

### Differential Radial Capillary Action of Ligand Assay (DRaCALA)

The DRaCALA assay was performed as described by Roelofs et al. [43]. Briefly, ^32^P-labelled c-di-GMP is generated from [α-^32^P]-GTP using purified WspR [83]. 20 μl of *E*. *coli* whole-cell lysates in binding buffer were mixed with 4nM ^32^P-labeled c-di-GMP. These mixtures were pipetted (2.5 μl) onto dry untreated nitrocellulose (GE Healthcare) in triplicate and allowed to dry for ten minutes. An FLA7100 Fujifilm Life Science PhosphorImager was used to detect luminescence following a 10-min exposure of blotted nitrocellulose to phosphorimager film. Data were quantified using Fujifilm Multi Gauge software v3.0.

### Preparation of whole cell lysates

BL21(DE3) pACDuet-1 containing strains were grown in LB medium until O.D._600_ of 0.6 and subsequently induced overnight with 1 mM IPTG at 18°C. 4 O.D. of bacteria were collected by centrifugation and suspended in 100 μl 40 mM Tris (pH 7.5), 100 mM NaCl, 10 mM MgCl2 binding buffer containing 2 mM PMSF, 20 μg/mL DNase, and 0.5 mg/mL lysozyme. Cells were lysed by three freeze/thaw cycles. Lysates were directly used in DRaCALA binding assays.

### Bacterial two-hybrid assay

Bacterial two hybrid experiments and cloning strategies were performed as previously described [17]. DNA fragments encoding proteins of interest were cloned into pKT25 and PUT18c. DNA regions encoding HptB, HsbA, HsbD and HsbR were amplified by using primers couples BTHhptBfw/BTHhptBrev, BTH43fw2/BTH43rev, BTH46fw/BTH46rev and BTH47fw/BTH47rev, respectively. HsbD PCR products were digested with XbaI and EcoRI and cloned into pKT25 (yielding T25-43s plasmid) while HptB, HsbA, HsbD and HsbR product were digested with XbaI and KpnI and cloned into PUT18c, yielding T18-hptB, T18-46, T18-43s and T18-47 plasmid, respectively. Recombinant pKT25 and pUT18C plasmids were transformed simultaneously into the *E*. *coli* DHM1 strain and transformants were spotted onto LB agar plates supplemented with 1mM isopropyl β-d-thiogalactoside (IPTG) in the presence of 100 μg/ml ampicillin, 50 μg/ml kanamycin, and 100 μg/ml 5-bromo-4-chloro-indolyl-β-d-galactopyranoside (X-gal). Positive interactions were identified as blue colonies after 24h incubation at 30°C and quantified by β-galactosidase assays. The positive controls used in the study were pUT18C and pKT25 derivatives encoding the leucine zipper from GCN4, which strongly dimerizes (zip).

### Transmission electron microscopy (TEM)

Log phase cultures (OD600 0.25 to 0.5) were spotted on a on a 400 mesh copper grid covered with Parlodion film, incubated for 15 min at room temperature, and then fixed in a surface-associated state with 0.1% glutaraldehyde. Preparations were then washed 3 times with water and negatively-stained twice with 1% uranyl acetate. Pictures were taken with a FEI Morgagni 268(D) electron microscope.

### Blot overlay

Cultures of *P*. *aeruginosa* PAK WT strain carrying plasmid for either HsbD (M2-tagged), SadC, HsbR overexpression or the empty vector were grown overnight at 37°C in presence of appropriate antibiotics. Cultures were normalized to the same O.D. and then 10 μl was spotted onto a nitrocellulose membrane (GE Healthcare). The spots were let to dry before the membrane was blocked with 5% milk in PBST (4 mM KH_2_PO_4_, 16 mM Na_2_HPO_4_, 115 mM NaCl (pH 7.4) and 0.05% Tween-20) for 1h at room temperature and then incubated overnight at 4C in 5% milk in PBST with 5ug of purified hemagglutinin-tagged HsbA (HsbA-HA) tagged. The next day, the membrane was washed three time with PBST to wash off unbound HsbA-HA. Membrane-bound HsbA-HA was detected by using monoclonal anti-HA antibody (Invitrogen) at a dilution 1:5000. Secondary antibody conjugated to horseradish peroxidase was used at a dilution of 1:5000. Western blots were developed using Super-Signal West Pico Chemiluminescent Substrate (Pierce) and visualized on a LAS3000 Fuji Imager. The same procedure was followed for the detection of HsbA_S56D_-HA and HsbA_S56A_-HA interaction with HsbD. HsbA-HA, HsbA_S56D_-HA, HsbA_S56A_-HA proteins were previously purified using anti-HA agarose beads and a HA-immunoprecipitation kit (Thermo Fisher Scientific). The protein content was analyzed by SDS-PAGE and Western blot using anti-HA (Invitrogen) and anti-FLAG M2 (Sigma) antibody.

### Construction of fluorescent fusions

To construct C-terminal YFP(Venus) fusions to *hsbD*, the gene was amplified by PCR with primers phsbD.1F- phsbD.2R (HsbD-YFP) or primers phsbD.3F- phsbD.2R and fragments were cloned into pME6032::Venus (IPTG inducible, see S1 and S2 Tables).

### Microscopy culture conditions

For microscopy, all strain containing fluorescent fusion(s) were grown overnight in LB presence of specific antibiotics. The next day, cell cultures were diluted to an O.D. 600 nm of 1.5 in 3ml LB with antibiotic(s) and 100μM IPTG±0.02% arabinose and they were grown at 37°C in glass tubes with shaking at 180 rpm for 2 hours.

### Fluorescence microscopy

Phase contrast and fluorescence microscopy were performed on a DeltaVision Core (Applied Precision, USA)/Olympus IX71 microscope equipped with an UPlanSApo 100×/1.40 Oil objective (Olympus, Japan) and a coolSNAP HQ-2 (Photometrics, USA) CCD camera. Cells were placed on a patch consisting of 1% agarose (Sigma, USA) in water (Sigma, USA). For time-lapse experiments the agarose patch contained LB agar, 0.02% arabinose, 100 μM of IPTG, 100 μg/ml tetracycline and 50 μg/ml of gentamycin. Images were processed with Image J (NIH, USA). Colocalization was analyzed using Cell Profiler cell image analysis software (Broad Institute) and a customized pipeline.

## Supporting Information

S1 Fig Schematic representation of the HptB cascade. (EPS)

S2 Fig Complementation of Δ*hptB*Δ*hsbD* mutant. Biofilm formation of *P*. *aeruginosa* strains measured by crystal violet staining. Bacterial strains were grown in LB medium in 24-microtiter plates for 14 hours in presence of 250 μg/ml carbenicillin.
(EPS)

S3 Fig RsmZ expression is not affected by the HptB/HsbD pathway. β-Galactosidase activity of a *rsmZ–lacZ* transcriptional fusion in PAK wild type (WT) and PAK Δ*hsbD*, Δ*hptB*, Δ*hptB*Δ*hsbD*, Δ*retS* and Δ*retS*Δ*hsbD* mutant strains grown in rich LB medium to an OD600~2.0, as described in Bordi *et al*. (2010). Each value is the average of three different cultures ± standard deviation (**, *p*< 0.01).
(EPS)

S4 Fig HsbA control of biofilm formation. Biofilm formation of *P*. *aeruginosa* wild-type PAK, Δ*hptB*, Δ*hptB*Δ*hsbD* and Δ*hptB*Δ*hsbA* strains measured by crystal violet staining. Bacterial strains were grown in LB medium in 24-microtiter plates for 14 hours. Each value is the average of three different cultures ± standard deviation.
(EPS)

S5 Fig HsbA control of swarming motility and role of HsbD. **(A-B)** Swarming motility of *P*. *aeruginosa* wild-type PAK, Δ*hsbA*, Δ*hsbD* and Δ*hsbA*Δ*hsbD* strains measured by surface area covered (cm^2^). Bacterial strains were grown in minimal medium supplemented with glucose and casamino-acids **(A)** or nutrient agar plus glucose **(B)**. **(C)** Swarming motility of *P*. *aeruginosa* Δ*hptB*, Δ*hptB*Δ*hsbD* and Δ*hptB*Δ*hsbA* strains grown in nutrient agar plus glucose. Surface area covered by the swarming cells (± standard deviation) was calculated by averaging data from four individual swarm plates (Asterisks indicate statistically significant difference **, *p* < 0.01).
(EPS)

S6 Fig Light microscopy images of twitching cells. Phase contrast images of the leading edge of A) PAK WT B) PAK Δ*hptB* C) PAK Δ*pilA* wild type and D) PAK Δ*hptB*Δ*hsbD* colony. Pictures were taken using Z1 Zeiss Axio Observer with 20X and were edited with ImageJ. Scale bar is illustrated in each figure.
(TIF)

S7 Fig HsbA control of twitching motility and role of HsbD. **(A)** Twitching motility of *P*. *aeruginosa* wild-type PAK, Δ*hsbA*, Δ*hsbD* and Δ*hsbA*Δ*hsbD* strains measured by surface area covered (cm^2^). Bacterial strains were expressing in *trans* either HsbA, HsbA_S56D_ or HsbA_S56A_ or the empty vector (ev: pME6032, see S1 Table). **(B)** Twitching motility of *P*. *aeruginosa* Δ*hptB*, Δ*hptB*Δ*hsbD* and Δ*hptB*Δ*hsbA* strains. Surface area covered by the twitching cells (± standard deviation) was calculated by averaging data from four individual swarm plates.
(EPS)

S8 Fig Functionality of HsbD-YFP and HsbD_Cter_-YFP fusions. **(A)** Expression of HsbD or HsbD_Cter_ -YFP fusion and detection of HsbD DGC activity by Congo red binding. **(B)** Twitching motility of a Δ*hptB*Δ*hsbD* mutant strain complemented with pME6032 vector expressing HsbD or HsbD_Cter_ -YFP fusions or empty vector (ev).
(EPS)

S9 Fig Localization dynamics of HsbD and FhlF. **(A)** Fluorescence images of HsbD-YFP and FhlF-RFP coexpressed in a *P*. *aeruginosa* PAK wild-type. **(B)** Localization of FhlF-RFP (red) and HsbD-YFP (green) in a *P*. *aeruginosa* wild-type strain. Scale bar = 1 μm. An overlay of the fluorescence channel(s) and the phase contrast image illustrate FlhF-RFP and HsbD-YFP subcellular localization pattern. Cartoon outline represents what observed in the panels. Scale bar = 1 μm.
(EPS)

S1 Table Strains and plasmids used in this study. (DOCX)

S2 Table List of primers used in this study. (DOCX)

S1 Movie TimeLapse microscopy of dividing *P*. *aeruginosa* PAK wild-type cells coexpressing in HsbD-YFP and FhlF-RFP. Overlay of fluorescence channels and phase contrast is shown.
(AVI)

S2 Movie TimeLapse microscopy of dividing *P*. *aeruginosa* PAK wild-type cells coexpressing in HsbD-YFP and FhlF-RFP. Overlay of fluorescence channels and phase contrast is shown.
(AVI)

## References

[1] McCormick K, Baillie GS. Compartmentalisation of second messenger signalling pathways. Curr Opin Genet Dev. 2014;27:20–5. Epub 2014/05/06. 10.1016/j.gde.2014.02.001 24791689

[2] Romling U, Galperin MY, Gomelsky M. Cyclic di-GMP: the first 25 years of a universal bacterial second messenger. Microbiol Mol Biol Rev. 2013;77(1):1–52. Epub 2013/03/09. 10.1128/MMBR.00043-12 23471616 PMC3591986

[3] Massie JP, Reynolds EL, Koestler BJ, Cong JP, Agostoni M, Waters CM. Quantification of high-specificity cyclic diguanylate signaling. Proc Natl Acad Sci U S A. 2012;109(31):12746–51. Epub 2012/07/18. 10.1073/pnas.1115663109 22802636 PMC3411991

[4] Haussler S. Biofilm formation by the small colony variant phenotype of *Pseudomonas aeruginosa*. Environ Microbiol. 2004;6(6):546–51. Epub 2004/05/15. 10.1111/j.1462-2920.2004.00618.x 15142242

[5] Jenal U, Malone J. Mechanisms of cyclic-di-GMP signaling in bacteria. Annu Rev Genet. 2006;40:385–407. Epub 2006/08/10. 10.1146/annurev.genet.40.110405.090423 16895465

[6] Chen ZH, Schaap P. The prokaryote messenger c-di-GMP triggers stalk cell differentiation in *Dictyostelium*. Nature. 2012;488(7413):680–3. Epub 2012/08/07. 10.1038/nature11313 22864416 PMC3939355

[7] Burdette DL, Monroe KM, Sotelo-Troha K, Iwig JS, Eckert B, Hyodo M, et al STING is a direct innate immune sensor of cyclic di-GMP. Nature. 2011;478(7370):515–8. Epub 2011/09/29. 10.1038/nature10429 21947006 PMC3203314

[8] Romling U, Amikam D. Cyclic di-GMP as a second messenger. Curr Opin Microbiol. 2006;9(2):218–28. Epub 2006/03/15. 10.1016/j.mib.2006.02.010 16530465

[9] Simm R, Morr M, Kader A, Nimtz M, Romling U. GGDEF and EAL domains inversely regulate cyclic di-GMP levels and transition from sessility to motility. Mol Microbiol. 2004;53(4):1123–34. Epub 2004/08/13. 10.1111/j.1365-2958.2004.04206.x 15306016

[10] Hengge R. Principles of c-di-GMP signalling in bacteria. Nat Rev Microbiol. 2009;7(4):263–73. Epub 2009/03/17. 10.1038/nrmicro2109 19287449

[11] Jenal U. Cyclic di-guanosine-monophosphate comes of age: a novel secondary messenger involved in modulating cell surface structures in bacteria? Curr Opin Microbiol. 2004;7(2):185–91. Epub 2004/04/06. 10.1016/j.mib.2004.02.007 15063857

[12] Mills E, Pultz IS, Kulasekara HD, Miller SI. The bacterial second messenger c-di-GMP: mechanisms of signalling. Cell Microbiol. 2011;13(8):1122–9. Epub 2011/06/29. 10.1111/j.1462-5822.2011.01619.x 21707905

[13] Galperin MY. A census of membrane-bound and intracellular signal transduction proteins in bacteria: bacterial IQ, extroverts and introverts. BMC Microbiol. 2005;5:35 Epub 2005/06/16. 10.1186/1471-2180-5-35 15955239 PMC1183210

[14] Christen M, Kulasekara HD, Christen B, Kulasekara BR, Hoffman LR, Miller SI. Asymmetrical distribution of the second messenger c-di-GMP upon bacterial cell division. Science. 2010;328(5983):1295–7. Epub 2010/06/05. 10.1126/science.1188658 20522779 PMC3906730

[15] Valentini M, Filloux A. Biofilms and c-di-GMP Signaling: Lessons from *Pseudomonas aeruginosa* and other Bacteria. J Biol Chem. 2016. 10.1074/jbc.R115.711507 PMC4933438 27129226

[16] Kulasakara H, Lee V, Brencic A, Liberati N, Urbach J, Miyata S, et al Analysis of *Pseudomonas aeruginosa* diguanylate cyclases and phosphodiesterases reveals a role for bis-(3'-5')-cyclic-GMP in virulence. Proc Natl Acad Sci U S A. 2006;103(8):2839–44. Epub 2006/02/16. 10.1073/pnas.0511090103 16477007 PMC1413825

[17] Bordi C, Lamy MC, Ventre I, Termine E, Hachani A, Fillet S, et al Regulatory RNAs and the HptB/RetS signalling pathways fine-tune *Pseudomonas aeruginosa* pathogenesis. Mol Microbiol. 2010;76(6):1427–43. Epub 2010/04/20. 10.1111/j.1365-2958.2010.07146.x 20398205 PMC2904497

[18] Hsu JL, Chen HC, Peng HL, Chang HY. Characterization of the histidine-containing phosphotransfer protein B-mediated multistep phosphorelay system in *Pseudomonas aeruginosa* PAO1. J Biol Chem. 2008;283(15):9933–44. Epub 2008/02/08. 10.1074/jbc.M708836200 18256026 PMC2442296

[19] Houot L, Fanni A, de Bentzmann S, Bordi C. A bacterial two-hybrid genome fragment library for deciphering regulatory networks of the opportunistic pathogen *Pseudomonas aeruginosa*. Microbiology. 2012;158(Pt 8):1964–71. Epub 2012/05/26. 10.1099/mic.0.057059-0 22628483

[20] Bhuwan M, Lee HJ, Peng HL, Chang HY. Histidine-containing phosphotransfer protein-B (HptB) regulates swarming motility through partner-switching system in *Pseudomonas aeruginosa* PAO1 strain. J Biol Chem. 2012;287(3):1903–14. Epub 2011/12/01. 10.1074/jbc.M111.256586 22128156 PMC3265871

[21] Lapouge K, Schubert M, Allain FH, Haas D. Gac/Rsm signal transduction pathway of gamma-proteobacteria: from RNA recognition to regulation of social behaviour. Mol Microbiol. 2008;67(2):241–53. Epub 2007/12/01. 10.1111/j.1365-2958.2007.06042.x 18047567

[22] Mikkelsen H, Sivaneson M, Filloux A. Key two-component regulatory systems that control biofilm formation in *Pseudomonas aeruginosa*. Environ Microbiol. 2011;13(7):1666–81. Epub 2011/05/11. 10.1111/j.1462-2920.2011.02495.x 21554516

[23] Ventre I, Goodman AL, Vallet-Gely I, Vasseur P, Soscia C, Molin S, et al Multiple sensors control reciprocal expression of *Pseudomonas aeruginosa* regulatory RNA and virulence genes. Proc Natl Acad Sci U S A. 2006;103(1):171–6. Epub 2005/12/24. 10.1073/pnas.0507407103 16373506 PMC1324988

[24] Moscoso JA, Mikkelsen H, Heeb S, Williams P, Filloux A. The *Pseudomonas aeruginosa* sensor RetS switches type III and type VI secretion via c-di-GMP signalling. Environ Microbiol. 2011;13(12):3128–38. Epub 2011/10/01. 10.1111/j.1462-2920.2011.02595.x 21955777

[25] Moscoso JA, Jaeger T, Valentini M, Hui K, Jenal U, Filloux A. The diguanylate cyclase SadC is a central player of the Gac/Rsm-mediated biofilm formation in *Pseudomonas aeruginosa*. J Bacteriol. 2014;196(23):4081–8. Epub 2014/09/17. 10.1128/JB.01850-14 25225264 PMC4248864

[26] Petrova OE, Sauer K. SagS contributes to the motile-sessile switch and acts in concert with BfiSR to enable *Pseudomonas aeruginosa* biofilm formation. J Bacteriol. 2011;193(23):6614–28. Epub 2011/09/29. 10.1128/JB.00305-11 21949078 PMC3232883

[27] Lin CT, Huang YJ, Chu PH, Hsu JL, Huang CH, Peng HL. Identification of an HptB-mediated multi-step phosphorelay in *Pseudomonas aeruginosa* PAO1. Res Microbiol. 2006;157(2):169–75. Epub 2005/09/27. 10.1016/j.resmic.2005.06.012 16182517

[28] Mern DS, Ha SW, Khodaverdi V, Gliese N, Gorisch H. A complex regulatory network controls aerobic ethanol oxidation in *Pseudomonas aeruginosa*: indication of four levels of sensor kinases and response regulators. Microbiology. 2010;156(Pt 5):1505–16. Epub 2010/01/23. 10.1099/mic.0.032847-0 20093290

[29] Winsor GL, Lam DK, Fleming L, Lo R, Whiteside MD, Yu NY, et al Pseudomonas Genome Database: improved comparative analysis and population genomics capability for *Pseudomonas* genomes. Nucleic Acids Res. 2011;39(Database issue):D596–600. Epub 2010/10/12. 10.1093/nar/gkq869 20929876 PMC3013766

[30] Garcia B, Latasa C, Solano C, Garcia-del Portillo F, Gamazo C, Lasa I. Role of the GGDEF protein family in *Salmonella* cellulose biosynthesis and biofilm formation. Mol Microbiol. 2004;54(1):264–77. Epub 2004/10/02. 10.1111/j.1365-2958.2004.04269.x 15458421

[31] Christen B, Christen M, Paul R, Schmid F, Folcher M, Jenoe P, et al Allosteric control of cyclic di-GMP signaling. J Biol Chem. 2006;281(42):32015–24. Epub 2006/08/23. 10.1074/jbc.M603589200 16923812

[32] Rybtke MT, Borlee BR, Murakami K, Irie Y, Hentzer M, Nielsen TE, et al Fluorescence-based reporter for gauging cyclic di-GMP levels in *Pseudomonas aeruginosa*. Appl Environ Microbiol. 2012;78(15):5060–9. Epub 2012/05/15. 10.1128/AEM.00414-12 22582064 PMC3416407

[33] Chan C, Paul R, Samoray D, Amiot NC, Giese B, Jenal U, et al Structural basis of activity and allosteric control of diguanylate cyclase. Proc Natl Acad Sci U S A. 2004;101(49):17084–9. Epub 2004/12/01. 10.1073/pnas.0406134101 15569936 PMC535365

[34] Daniels R, Vanderleyden J, Michiels J. Quorum sensing and swarming migration in bacteria. FEMS Microbiol Rev. 2004;28(3):261–89. Epub 2004/09/29. 15449604 10.1016/j.femsre.2003.09.004

[35] Kohler T, Curty LK, Barja F, van Delden C, Pechere JC. Swarming of *Pseudomonas aeruginosa* is dependent on cell-to-cell signaling and requires flagella and pili. J Bacteriol. 2000;182(21):5990–6. Epub 2000/10/13. 11029417 10.1128/jb.182.21.5990-5996.2000 PMC94731

[36] Murray TS, Kazmierczak BI. *Pseudomonas aeruginosa* exhibits sliding motility in the absence of type IV pili and flagella. J Bacteriol. 2008;190(8):2700–8. Epub 2007/12/11. 10.1128/JB.01620-07 18065549 PMC2293233

[37] Kuchma SL, Griffin EF, O'Toole GA. Minor pilins of the type IV pilus system participate in the negative regulation of swarming motility. J Bacteriol. 2012;194(19):5388–403. Epub 2012/08/07. 10.1128/JB.00899-12 22865844 PMC3457191

[38] Kuchma SL, Ballok AE, Merritt JH, Hammond JH, Lu W, Rabinowitz JD, et al Cyclic-di-GMP-mediated repression of swarming motility by *Pseudomonas aeruginosa*: the pilY1 gene and its impact on surface-associated behaviors. J Bacteriol. 2010;192(12):2950–64. Epub 2010/03/18. 10.1128/JB.01642-09 20233936 PMC2901681

[39] Anyan ME, Amiri A, Harvey CW, Tierra G, Morales-Soto N, Driscoll CM, et al Type IV pili interactions promote intercellular association and moderate swarming of *Pseudomonas aeruginosa*. Proc Natl Acad Sci U S A. 2014;111(50):18013–8. Epub 2014/12/04. 10.1073/pnas.1414661111 25468980 PMC4273417

[40] Zolfaghar I, Angus AA, Kang PJ, To A, Evans DJ, Fleiszig SM. Mutation of retS, encoding a putative hybrid two-component regulatory protein in *Pseudomonas aeruginosa*, attenuates multiple virulence mechanisms. Microbes Infect. 2005;7(13):1305–16. Epub 2005/07/20. 10.1016/j.micinf.2005.04.017 16027020

[41] Nesper J, Reinders A, Glatter T, Schmidt A, Jenal U. A novel capture compound for the identification and analysis of cyclic di-GMP binding proteins. J Proteomics. 2012;75(15):4874–8. Epub 2012/06/02. 10.1016/j.jprot.2012.05.033 22652488

[42] Duvel J, Bertinetti D, Moller S, Schwede F, Morr M, Wissing J, et al A chemical proteomics approach to identify c-di-GMP binding proteins in *Pseudomonas aeruginosa*. J Microbiol Methods. 2012;88(2):229–36. Epub 2011/12/20. 10.1016/j.mimet.2011.11.015 22178430

[43] Roelofs KG, Wang J, Sintim HO, Lee VT. Differential radial capillary action of ligand assay for high-throughput detection of protein-metabolite interactions. Proc Natl Acad Sci U S A. 2011;108(37):15528–33. Epub 2011/08/31. 10.1073/pnas.1018949108 21876132 PMC3174574

[44] Baker AE, Diepold A, Kuchma SL, Scott JE, Ha DG, Orazi G, et al PilZ Domain Protein FlgZ Mediates Cyclic Di-GMP-Dependent Swarming Motility Control in *Pseudomonas aeruginosa*. J Bacteriol. 2016;198(13):1837–46. Epub 2016/04/27. 10.1128/JB.00196-16 27114465 PMC4907108

[45] Martinez-Granero F, Navazo A, Barahona E, Redondo-Nieto M, Gonzalez de Heredia E, Baena I, et al Identification of *flgZ* as a flagellar gene encoding a PilZ domain protein that regulates swimming motility and biofilm formation in Pseudomonas. PloS one. 2014;9(2):e87608 Epub 2014/02/08. 10.1371/journal.pone.0087608 24504373 PMC3913639

[46] Pandza S, Baetens M, Park CH, Au T, Keyhan M, Matin A. The G-protein FlhF has a role in polar flagellar placement and general stress response induction in *Pseudomonas putida*. Mol Microbiol. 2000;36(2):414–23. Epub 2000/05/03. 10792727 10.1046/j.1365-2958.2000.01859.x

[47] Kazmierczak BI, Hendrixson DR. Spatial and numerical regulation of flagellar biosynthesis in polarly flagellated bacteria. Mol Microbiol. 2013;88(4):655–63. Epub 2013/04/23. 10.1111/mmi.12221 23600726 PMC3654036

[48] Cowles KN, Moser TS, Siryaporn A, Nyakudarika N, Dixon W, Turner JJ, et al The putative Poc complex controls two distinct *Pseudomonas aeruginosa* polar motility mechanisms. Mol Microbiol. 2013;90(5):923–38. Epub 2013/10/10. 10.1111/mmi.12403 24102920 PMC4666538

[49] Burrows LL. A new route for polar navigation. Mol Microbiol. 2013;90(5):919–22. Epub 2013/10/19. 10.1111/mmi.12433 24134731

[50] Coggan KA, Wolfgang MC. Global regulatory pathways and cross-talk control *Pseudomonas aeruginosa* environmental lifestyle and virulence phenotype. Current issues in molecular biology. 2012;14(2):47–70. Epub 2012/02/23. 22354680 PMC12747716

[51] Starnbach MN, Lory S. The fliA (rpoF) gene of Pseudomonas aeruginosa encodes an alternative sigma factor required for flagellin synthesis. Mol Microbiol. 1992;6(4):459–69. Epub 1992/02/01. 1560774 10.1111/j.1365-2958.1992.tb01490.x

[52] Dasgupta N, Wolfgang MC, Goodman AL, Arora SK, Jyot J, Lory S, et al A four-tiered transcriptional regulatory circuit controls flagellar biogenesis in Pseudomonas aeruginosa. Mol Microbiol. 2003;50(3):809–24. Epub 2003/11/18. 14617143 10.1046/j.1365-2958.2003.03740.x

[53] Lo YL, Shen L, Chang CH, Bhuwan M, Chiu CH, Chang HY. Regulation of Motility and Phenazine Pigment Production by FliA Is Cyclic-di-GMP Dependent in Pseudomonas aeruginosa PAO1. PloS one. 2016;11(5):e0155397 Epub 2016/05/14. 10.1371/journal.pone.0155397 27175902 PMC4866697

[54] Hickman JW, Tifrea DF, Harwood CS. A chemosensory system that regulates biofilm formation through modulation of cyclic diguanylate levels. Proc Natl Acad Sci U S A. 2005;102(40):14422–7. Epub 2005/09/28. 10.1073/pnas.0507170102 16186483 PMC1234902

[55] Paul R, Weiser S, Amiot NC, Chan C, Schirmer T, Giese B, et al Cell cycle-dependent dynamic localization of a bacterial response regulator with a novel di-guanylate cyclase output domain. Genes Dev. 2004;18(6):715–27. Epub 2004/04/13. 10.1101/gad.289504 15075296 PMC387245

[56] Ha DG, O'Toole GA. c-di-GMP and its Effects on Biofilm Formation and Dispersion: a Pseudomonas Aeruginosa Review. Microbiol Spectr. 2015;3(2):MB-0003-2014. Epub 2015/06/25. 10.1128/microbiolspec.MB-0003-2014 PMC4498269 26104694

[57] Conrad JC, Gibiansky ML, Jin F, Gordon VD, Motto DA, Mathewson MA, et al Flagella and pili-mediated near-surface single-cell motility mechanisms in P. aeruginosa. Biophys J. 2011;100(7):1608–16. Epub 2011/04/06. 10.1016/j.bpj.2011.02.020 21463573 PMC3072661

[58] Guvener ZT, Harwood CS. Subcellular location characteristics of the *Pseudomonas aeruginosa* GGDEF protein, WspR, indicate that it produces cyclic-di-GMP in response to growth on surfaces. Mol Microbiol. 2007;66(6):1459–73. Epub 2007/11/22. 10.1111/j.1365-2958.2007.06008.x 18028314 PMC4105145

[59] Russell MH, Bible AN, Fang X, Gooding JR, Campagna SR, Gomelsky M, et al Integration of the second messenger c-di-GMP into the chemotactic signaling pathway. MBio. 2013;4(2):e00001–13. Epub 2013/03/21. 10.1128/mBio.00001-13 23512960 PMC3604760

[60] Merritt JH, Ha DG, Cowles KN, Lu W, Morales DK, Rabinowitz J, et al Specific control of *Pseudomonas aeruginosa* surface-associated behaviors by two c-di-GMP diguanylate cyclases. MBio. 2010;1(4). Epub 2010/10/28. 10.1128/mBio.00183-10 PMC2957078 20978535

[61] Paul R, Weiser S, Amiot NC, Chan C, Schirmer T, Giese B, et al Cell cycle-dependent dynamic localization of a bacterial response regulator with a novel di-guanylate cyclase output domain. Genes Dev. 2004;18(6):715–27. Epub 2004/04/13. 10.1101/gad.289504 15075296 PMC387245

[62] Aldridge P, Paul R, Goymer P, Rainey P, Jenal U. Role of the GGDEF regulator PleD in polar development of *Caulobacter crescentus*. Mol Microbiol. 2003;47(6):1695–708. Epub 2003/03/08. 12622822 10.1046/j.1365-2958.2003.03401.x

[63] Lori C, Ozaki S, Steiner S, Bohm R, Abel S, Dubey BN, et al Cyclic di-GMP acts as a cell cycle oscillator to drive chromosome replication. Nature. 2015;523(7559):236–9. Epub 2015/05/07. 10.1038/nature14473 25945741

[64] Suzuki T, Iino T. Isolation and characterization of multiflagellate mutants of *Pseudomonas aeruginosa*. J Bacteriol. 1980;143(3):1471–9. Epub 1980/09/01. 6773930 10.1128/jb.143.3.1471-1479.1980 PMC294538

[65] Kulasekara BR, Kamischke C, Kulasekara HD, Christen M, Wiggins PA, Miller SI. c-di-GMP heterogeneity is generated by the chemotaxis machinery to regulate flagellar motility. Elife. 2013;2:e01402 Epub 2013/12/19. 10.7554/eLife.01402 24347546 PMC3861689

[66] Merritt JH, Brothers KM, Kuchma SL, O'Toole GA. SadC reciprocally influences biofilm formation and swarming motility via modulation of exopolysaccharide production and flagellar function. J Bacteriol. 2007;189(22):8154–64. Epub 2007/06/26. 10.1128/JB.00585-07 17586642 PMC2168701

[67] Sezonov G, Joseleau-Petit D, D'Ari R. *Escherichia coli* physiology in Luria-Bertani broth. J Bacteriol. 2007;189(23):8746–9. Epub 2007/10/02. 10.1128/JB.01368-07 17905994 PMC2168924

[68] Miller JH. Experiments in molecular genetics Cold Spring Harbor Laboratory, Cold Spring Harbor, NY 1972.

[69] Pruitt KD, Brown GR, Hiatt SM, Thibaud-Nissen F, Astashyn A, Ermolaeva O, et al RefSeq: an update on mammalian reference sequences. Nucleic Acids Res. 2014;42(Database issue):D756–63. Epub 2013/11/22. 10.1093/nar/gkt1114 24259432 PMC3965018

[70] Edgar RC. MUSCLE: multiple sequence alignment with high accuracy and high throughput. Nucleic Acids Res. 2004;32(5):1792–7. Epub 2004/03/23. 10.1093/nar/gkh340 15034147 PMC390337

[71] Saitou N, Nei M. The neighbor-joining method: a new method for reconstructing phylogenetic trees. Mol Biol Evol. 1987;4(4):406–25. Epub 1987/07/01. 3447015 10.1093/oxfordjournals.molbev.a040454

[72] Tamura K, Stecher G, Peterson D, Filipski A, Kumar S. MEGA6: Molecular Evolutionary Genetics Analysis version 6.0. Mol Biol Evol. 2013;30(12):2725–9. Epub 2013/10/18. 10.1093/molbev/mst197 24132122 PMC3840312

[73] Zuckerkandl E, Pauling L. Molecules as documents of evolutionary history. J Theor Biol. 1965;8(2):357–66. Epub 1965/03/01. 5876245 10.1016/0022-5193(65)90083-4

[74] Sambrook J, Fritsch E. F., and Maniatis T. Molecular cloning: a laboratory manual 2nd ed. ed. Press neCSHL, editor. 2nd ed. Cold Spring Harbor Laboratory Press Cold Spring Harbor, NY 1989.

[75] Pessi G, Haas D. Transcriptional control of the hydrogen cyanide biosynthetic genes hcnABC by the anaerobic regulator ANR and the quorum-sensing regulators LasR and RhlR in *Pseudomonas aeruginosa*. J Bacteriol. 2000;182(24):6940–9. Epub 2000/11/28. 11092854 10.1128/jb.182.24.6940-6949.2000 PMC94819

[76] Rashid MH, Kornberg A. Inorganic polyphosphate is needed for swimming, swarming, and twitching motilities of *Pseudomonas aeruginosa*. Proc Natl Acad Sci U S A. 2000;97(9):4885–90. Epub 2000/04/12. 10.1073/pnas.060030097 10758151 PMC18327

[77] Mikkelsen H, Ball G, Giraud C, Filloux A. Expression of *Pseudomonas aeruginosa* CupD fimbrial genes is antagonistically controlled by RcsB and the EAL-containing PvrR response regulators. PloS one. 2009;4(6):e6018 Epub 2009/06/24. 10.1371/journal.pone.0006018 19547710 PMC2696094

[78] Wenner N, Maes A, Cotado-Sampayo M, Lapouge K. NrsZ: a novel, processed, nitrogen-dependent, small non-coding RNA that regulates *Pseudomonas aeruginosa* PAO1 virulence. Environ Microbiol. 2014;16(4):1053–68. Epub 2013/12/07. 10.1111/1462-2920.12272 24308329 PMC4253122

[79] Caiazza NC, Merritt JH, Brothers KM, O'Toole GA. Inverse regulation of biofilm formation and swarming motility by *Pseudomonas aeruginosa* PA14. J Bacteriol. 2007;189(9):3603–12. Epub 2007/03/06. 10.1128/JB.01685-06 17337585 PMC1855903

[80] Whitchurch CB, Leech AJ, Young MD, Kennedy D, Sargent JL, Bertrand JJ, et al Characterization of a complex chemosensory signal transduction system which controls twitching motility in *Pseudomonas aeruginosa*. Mol Microbiol. 2004;52(3):873–93. Epub 2004/04/23. 10.1111/j.1365-2958.2004.04026.x 15101991

[81] Mazumder R, Phelps TJ, Krieg NR, Benoit RE. Determining chemotactic responses by two subsurface microaerophiles using a simplified capillary assay method. J Microbiol Methods. 1999;37(3):255–63. Epub 1999/09/10. 10480269 10.1016/s0167-7012(99)00072-x

[82] Spangler C BA, Jenal U, Seifert R, Kaever V. A liquid chromatography-coupled tandem mass spectrometry method for quantitation of cyclic di-guanosine monophosphate. J Microbiol Methods. 2010;81(3):226–31. Epub 2010/04/10. 10.1016/j.mimet.2010.03.020 20385176

[83] Lieberman OJ, Orr MW, Wang Y, Lee VT. High-throughput screening using the differential radial capillary action of ligand assay identifies ebselen as an inhibitor of diguanylate cyclases. ACS Chem Biol. 2014;9(1):183–92. Epub 2013/10/19. 10.1021/cb400485k 24134695 PMC4545405

