# Correction: The Diguanylate Cyclase HsbD Intersects with the HptB Regulatory Cascade to Control *Pseudomonas aeruginosa* Biofilm and Motility

**DOI:** 10.1371/journal.pgen.1006473

**Published:** 2016-11-30

**Authors:** Martina Valentini, Benoît-Joseph Laventie, Joana A. Moscoso, Urs Jenal, Alain Filloux

The third author's name is incorrect. The correct name is: Joana A. Moscoso. The correct citation is: Valentini M, Laventie B-J, Moscoso JA, Jenal U, Filloux A (2016) The Diguanylate Cyclase HsbD Intersects with the HptB Regulatory Cascade to Control *Pseudomonas aeruginosa* Biofilm and Motility. PLoS Genet 12(10): e1006354. doi:10.1371/journal.pgen.1006354. In the Author Contributions section, the initials JM should appear as JAM.

## References

[1] Valentini M, Laventie B-J, Moscoso J, Jenal U, Filloux A (2016) The Diguanylate Cyclase HsbD Intersects with the HptB Regulatory Cascade to Control *Pseudomonas aeruginosa* Biofilm and Motility. PLoS Genet 12(10): e1006354 doi:10.1371/journal.pgen.1006354 27792789 10.1371/journal.pgen.1006354 PMC5085249

