# Supplementary figures and images for: The Diguanylate Cyclase HsbD Intersects with the HptB Regulatory Cascade to Control Pseudomonas aeruginosa Biofilm and Motility
Source: PLoS Genet. 2016 Oct 28;12(10):e1006354. doi: 10.1371/journal.pgen.1006354 (PMC5085249; doi:10.1371/journal.pgen.1006354)

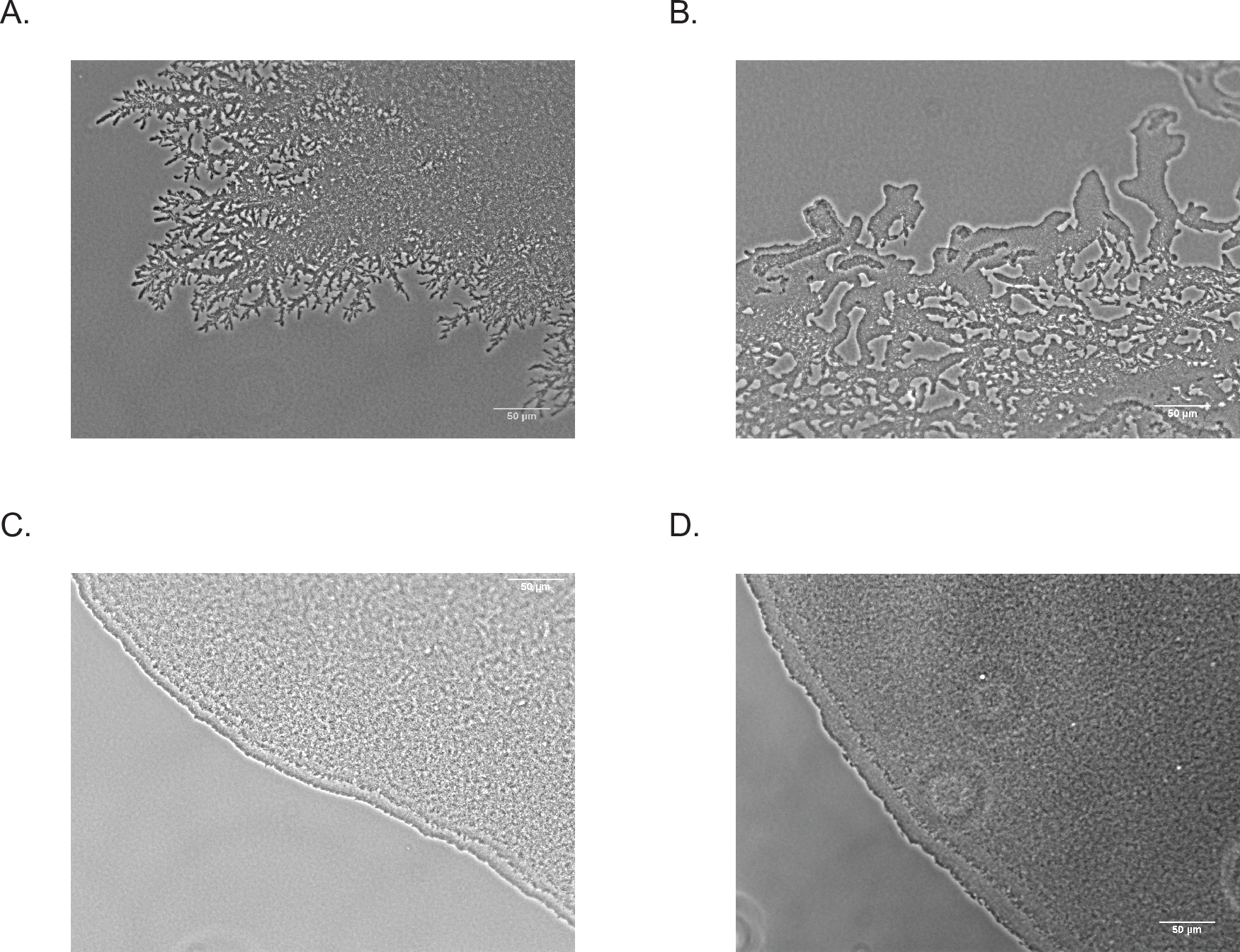

Supplement: S6 Fig — Phase contrast images of the leading edge of A) PAK WT B) PAK ΔhptB C) PAK ΔpilA wild type and D) PAK ΔhptBΔhsbD colony. Pictures were taken using Z1 Zeiss Axio Observer with 20X and were edited with ImageJ. Scale bar is illustrated in each figure. (TIF) [file pgen.1006354.s006.tif]
