# Supplementary material for: The Diguanylate Cyclase HsbD Intersects with the HptB Regulatory Cascade to Control Pseudomonas aeruginosa Biofilm and Motility
Source: PLoS Genet. 2016 Oct 28;12(10):e1006354. doi: 10.1371/journal.pgen.1006354 (PMC5085249; doi:10.1371/journal.pgen.1006354)
Supplement: S1 Table — (DOCX) [file pgen.1006354.s010.docx]

**Table S1** Strains and plasmids used in this study.

| Strains | Genotype/relevant characteristics | Source |
| --- | --- | --- |
| *E.coli* |  |  |
| BL21(DE3) | *F ompT hsdS_B_(*r_B_ m_B_*) gal dcm* (λDE3) | Novagen |
| DH5α | *recA1 endA1 hsdR17 supE44 thi-1 gyrA96 relA1* Δ(*lacZYA-argF*)*U169* [Φ80d*lacZ*M*15*]F^-^ NaI^r^ | [1] |
| DHM1 | Reporter strain for two-hybrid system (F−,*glnV44(AS), recA1, endA1, gyrA96 (Nal* r *), thi1,hsdR17*, *spoT1*, *rfbD1, cya*) | [2] |
| HB101 | *proA2 hsdS20*(r_B_^-^ m_B_^-^) *recA13 ara-14 lacYI galK2 rpsL20 supE44 xyl-5 mtl-1* F^-^ | [1] |
| S17-1/λ*pir* | *pro thi hsdR recA* chromosome::RP4-2 Tc::Mu Km::Tn*7*/λ*pir*; Tp^r^ Sm^r^ | [3,4] |
| TOP10 | F^-^ *mcr*A Δ(*mrr-hsd*RMS-*mcr*BC) φ80*lac*ZΔM15 Δ*lac*Χ74 *rec*A1 *ara*D139 Δ(*ara-leu*) 7697 *gal*U *gal*K *rps*L (Str^R^) *end*A1 *nup*G λ^-^ | Invitrogen |
| *P. aeruginosa* |  |  |
| PAK | Wild-type | [5] |
| PAK∆*hptB* | PAK with a *hptB* deletion | [6] |
| PAK∆*hsbA* | PAK with a *hsbA* deletion | [6] |
| PAK∆*hsbD* | PAK with a *hsbD* deletion | This study |
| PAK∆*hsbA*∆*hsbD* | PAK with deletion of *hsbA* and *hsbD* | This study |
| PAK∆*retS* | PAK with a *retS* deletion | [5] |
| PAK∆*rsmA* | PAK with a *rsmA* deletion | [7] |
| PAKΔ*hptB∆hsbD* | PAK with deletion of *hptB* and *hsbD* | This study |
| PAKΔ*hptB∆hsbA* | PAK with deletion of *hptB* and *hsbA* | [6] |
| PAKΔ*retS∆hsbD* | PAK with deletion of *retS* and *hsbD* | This study |
| PT712 | rhlA::Ω-Gm | [8] |
| Plasmids |  |  |
| pBBR1MCS-4 | Broad host range vector, Ap^R^ | [9] |
| pBBR1MCS-4-*hsbR* | *hsbR* cloned into pBBR1MCS-4 | [6] |
| pBBR1MCS-4-*hsbD* | *hsbD* cloned into pBBR1MCS-4 | This study |
| pBBR1MCS-4-*sadC* | *sadC* cloned into pBBR1MCS-4 | [10] |
| P_cdrA_-*gfp* | Plasmid expressing *gfp* from the promoter of *cdrA*, Ap^R^, Gm^R^ | [11] |
| pCR2.1-TA | Cloning plasmid, Ap^R^, Km^R^ | Invitrogen |
| pACYCDuet-1 | Expression vector used for expression of N-terminal 6-histidine tagged proteins, Cm^R^ | Novagen |
| pACYC-PA3343 | pACYCDuet-1 expressing PA3343_C-ter_ with an N-terminal histidine tag, Km^R^ | This study |
| pACYC-PA3343_a-site_ | pACYCDuet-1 expressing PA3343_C-ter_ with mutation in the active GGEEF site (GGAAF) and an N-terminal histidine tag, Km^R^ | This study |
| pACYC-PA3343_i-site_ | pACYCDuet-1 expressing PA3343_C-ter_ with mutation in the inhibitory RxxD site (AxxD) and an N-terminal histidine tag, Km^R^ | This study |
| pKNG101 | Suicide vector carrying *sacB*, Sm^R^ | [12] |
| pKNG101-∆*PA3343* | Mutator fragment for deletion of PA3343 (*hsbD*) (from amino acid 2 to 367), Sm^R^ | This study |
| pKT25 | Two-hybrid plasmid, *cyaAT25* fusion, Km^R^ | [2] |
| pUT18C | Two-hybrid plasmid, *cyaAT18* fusion, Ap^R^ | [2] |
| pME6032 | *lacI^Q^* P*_tac_* expression vector, Gm^R^ | [13] |
| pHsbD-FLAG | pME6032 expressing HsbD_C-ter_ with a C-terminal FLAG tag, Tc^R^ | This study |
| pHsbA-HA | pME6032 expressing *hsbA* with a C-terminal HA tag, Gm^R^ | This study |
| pHsbA_S56D_-HA | pME6032 expressing *hsbA_AGC🡪GAC_* with a C-terminal HA tag, Gm^R^ | This study |
| pHsbA_S56A_-HA | pME6032 expressing *hsbA_AGC🡪GGC_* with a C-terminal HA tag, Gm^R^ | This study |
| pME6032::VenusC | pME6032 based plasmid for fusion of Venus in C-terminal, Tc^R^ | Jenal lab |
| pHsbD-YFP | pME6032 expressing HsbD-YFP, Tc^R^ | This study |
| pHsbD_C-ter_-YFP | pME6032 expressing HsbD_C-ter_ -YFP, Tc^R^ | This study |
| FhlF-RFP | pJN105::FhlF-RFP, P*_BAD_*, Gm^R^ | [14] |
| pRK2013 | Helper plasmid; Tra^+^ Km^r^ | [1] |
| *rsmY-lacZ* | *rsmY-lacZ* transcriptional fusion | (5) |
| *rsmZ-lacZ* | *rsmZ-lacZ* transcriptional fusion | (5) |
| T25-HsbDs | Fusion of *hsbD* to *cya* gene T25 fragment in pKT25, Km^R^ | This study |
| T18-HsbDs | Fusion of *hsbD* to *cya* gene T18 fragment in pUT18C, Ap^R^ | This study |
| T18-HptB | Fusion of *hptB* to *cya* gene T18 fragment in pUT18C, Ap^R^ | This study |
| T18-HsbR | Fusion of *hsbR* to *cya* gene T18 fragment in pUT18C, Ap^R^ | This study |
| T18-HsbA | Fusion of *hsbA* to *cya* gene T18 fragment in pUT18C, Ap^R^ | This study |
| T18-HsbA_S56D_ | Fusion of *hsbA_AGC🡪GAC_* to *cya* gene T18 fragment in pUT18C (HsbA_S56D_), Ap^R^ | This study |
| T18-HsbA_S56A_ | Fusion of *hsbA_AGC🡪GGC_* to *cya* gene T18 fragment in pUT18C (HsbA_S56A_), Ap^R^ | This study |

**Additional references**

1. Sambrook J, E. F. Fritsch, and T. Maniatis (1989) Molecular cloning: a laboratory manual; Press neCSHL, editor. 2nd ed. Cold Spring Harbor Laboratory Press. Cold Spring Harbor, NY.

2. Karimova G, Ullmann A, Ladant D (2000) *Bordetella pertussis* adenylate cyclase toxin as a tool to analyze molecular interactions in a bacterial two-hybrid system. Int J Med Microbiol 290: 441-445.

3. Miller VL, Mekalanos JJ (1988) A novel suicide vector and its use in construction of insertion mutations: osmoregulation of outer membrane proteins and virulence determinants in Vibrio cholerae requires toxR. J Bacteriol 170: 2575-2583.

4. Simon R, O'Connell M, Labes M, Puhler A (1986) Plasmid vectors for the genetic analysis and manipulation of rhizobia and other gram-negative bacteria. Methods Enzymol 118: 640-659.

5. Goodman AL, Merighi M, Hyodo M, Ventre I, Filloux A, et al. (2009) Direct interaction between sensor kinase proteins mediates acute and chronic disease phenotypes in a bacterial pathogen. Genes Dev 23: 249-259.

6. Bordi C, Lamy MC, Ventre I, Termine E, Hachani A, et al. (2010) Regulatory RNAs and the HptB/RetS signalling pathways fine-tune *Pseudomonas aeruginosa* pathogenesis. Mol Microbiol 76: 1427-1443.

7. Ventre I, Goodman AL, Vallet-Gely I, Vasseur P, Soscia C, et al. (2006) Multiple sensors control reciprocal expression of *Pseudomonas aeruginosa* regulatory RNA and virulence genes. Proc Natl Acad Sci U S A 103: 171-176.

8. Kohler T, Curty LK, Barja F, van Delden C, Pechere JC (2000) Swarming of *Pseudomonas aeruginosa* is dependent on cell-to-cell signaling and requires flagella and pili. J Bacteriol 182: 5990-5996.

9. Kovach ME, Elzer PH, Hill DS, Robertson GT, Farris MA, et al. (1995) Four new derivatives of the broad-host-range cloning vector pBBR1MCS, carrying different antibiotic-resistance cassettes. Gene 166: 175-176.

10. Moscoso JA, Jaeger T, Valentini M, Hui K, Jenal U, et al. (2014) The diguanylate cyclase SadC is a central player in Gac/Rsm-mediated biofilm formation in *Pseudomonas aeruginosa*. J Bacteriol 196: 4081-4088.

11. Rybtke MT, Borlee BR, Murakami K, Irie Y, Hentzer M, et al. (2012) Fluorescence-based reporter for gauging cyclic di-GMP levels in *Pseudomonas aeruginosa*. Appl Environ Microbiol 78: 5060-5069.

12. Kaniga K, Delor I, Cornelis GR (1991) A wide-host-range suicide vector for improving reverse genetics in gram-negative bacteria: inactivation of the blaA gene of Yersinia enterocolitica. Gene 109: 137-141.

13. Heeb S, Blumer C, Haas D (2002) Regulatory RNA as mediator in GacA/RsmA-dependent global control of exoproduct formation in Pseudomonas fluorescens CHA0. Journal of Bacteriology 184: 1046-1056.

14. Cowles KN, Moser TS, Siryaporn A, Nyakudarika N, Dixon W, et al. (2013) The putative Poc complex controls two distinct Pseudomonas aeruginosa polar motility mechanisms. Molecular Microbiology 90: 923-938.
