# Supplementary material for: The Diguanylate Cyclase HsbD Intersects with the HptB Regulatory Cascade to Control Pseudomonas aeruginosa Biofilm and Motility
Source: PLoS Genet. 2016 Oct 28;12(10):e1006354. doi: 10.1371/journal.pgen.1006354 (PMC5085249; doi:10.1371/journal.pgen.1006354)
Supplement: S2 Table — (DOCX) [file pgen.1006354.s011.docx]

**Table S2.** List of primers used in this study.

| Name | Sequence^a^ (5’ → 3’) | Restriction site^#^ |
| --- | --- | --- |
| BTH43fw2 | GCTCTAGAGTTCAAGTCGCGCGAGCA | XbaI |
| BTH43rev | GGGAATTCACTCAGGCCACCACCTGGTT | EcoRI |
| BTH46fw | GCTCTAGATGAGATGAATCCCCCGGCGG | XbaI |
| BTH46rev | CCGAGCTCCGACGAAAGCGCGATTATGCCTGAGG | SacI |
| BTH47fw | GCTCTAGAGATGGCCATCACTGCGCTGCC | XbaI |
| BTH47rev | GGGGTACCTCAGCTGATCTTGAACAACTGC | KpnI |
| BTHhptBfw | GCTCTAGAGCGAATGTCCGCGCCGCATCTCGATGATCGTG | XbaI |
| BTHhtpBrev | GGGCCGGTACCTTGTCGCCGGAAAGGACGAAAACCTC | KpnI |
| pPA3343.1 | TGGCCCAGGTCAGCGGGGTCG |  |
| pPA3343.2 | TCAGGCCACCACGCACACCTCTTCTCT |  |
| pPA3343.3 | GTGTGCGTGGTGGCCTGAGTCCATGGC |  |
| pPA3343.4 | CCTGGCGCAGTTCGCAGACG |  |
| pPA3343.5 | GCTGCGCCGCGACCTCAAACC |  |
| pPA3343.6 | CGTGATGGGCGAAGGCGTCC |  |
| pHsbAS56D.fw | GCCACTTACCTGGACGACTCGGCCCTCGGCATG |  |
| pHsbAS56D.rev | CATGCCGAGGGCCGAGTCGTCCAGGTAAGTGGC |  |
| pHsbAS56A.fw | GCCACTTACCTGGACGGCTCGGCCCTCGGCATG |  |
| pHsbAS56A.rev | CATGCCGAGGGCCGAGCCGTCCAGGTAAGTGGC |  |
| pHsbD-YFP.1 | CCGAATTCGTGTGCGTGACACAGAAGGAC | EcoRI |
| pHsbD-YFP.2 | CGCTCGAGGGCCACCACCTGGTTACGGCC | XhoI |
| pHsbD-YFP.3 | CCGAATTCGTGTTCAAGTCGCGCGAGCATTTC | EcoRI |
| pHsbA-HAfw | CCGAATTCATGGCCATCACTGCGCTGCCC | EcoRI |
| pHsbA-HArev | CCCTCGAGGGCGTAGTCCGGCACGTCGTACGGGTAGCTGATCTTGAACAACTGCTC | XhoI |
| HsbD_i-site mut fw | GAGGCGAATGCCAGGGCTCCGCTGGACATGGCG |  |
| HsbD_i-site mut rev | CGCCATGTCCAGCGGAGCCCTGGCATTCGCCTC |  |
| HsbD_active-site mut fw | GTGCGCCTGGGCGGCGCAGCGTTCGCCCTGTTGCTG |  |
| HsbD_active-site mut rev | CAGCAACAGGGCGAACGCTGCGCCGCCCAGGCGCAC |  |
| pHsbD-His.1 | GGAATTCGTGCGTGACACAGAAGGACCGG | EcoRI |
| pHsbD-His.2 | CCCAAGCTTTCAGGCCACCACCTGGTTACG | HindIII |
| pHsbD-His.3 | GGAATTCGTTCAAGTCGCGCGAGCATTTC | EcoRI |

^#^restriction sites underlined
